# LCP1 upregulation via EGFR signaling promotes oral cancer progression through the JAK2/STAT3/IL-1β axis

**DOI:** 10.1186/s12935-025-03970-0

**Published:** 2025-10-03

**Authors:** Chiao-Rou Liu, Chia-Yu Yang, Kai-Ping Chang, Xiu-Ya Chan, Chu-Mi Hung, Kuan-Ming Lai, Hao-Ping Liu, Chih-Ching Wu

**Affiliations:** 1 Graduate Institute of Biomedical Sciences, College of Medicine, Chang Gung University, Taoyuan, Taiwan; 2 Department of Medical Biotechnology and Laboratory Science, College of Medicine, Chang Gung University, Taoyuan, 33302 Taiwan; 3 Department of Otolaryngology-Head and Neck Surgery, Linkou Chang Gung Memorial Hospital, Taoyuan, Taiwan; 4 Molecular Medicine Research Center, Chang Gung University, Taoyuan, Taiwan; 5 Department of Microbiology and Immunology, College of Medicine, Chang Gung University, Taoyuan, Taiwan; 6 Program in Translational Medicine, National Chung Hsing University, Taichung, Taiwan; 7 Hemato-Oncology Division Department of Internal Medicine, Changhua Christian Hospital, Changhua, Taiwan; 8 Department of Veterinary Medicine, College of Veterinary Medicine, National Chung Hsing University, Taichung, Taiwan; 9 Biotechnology Center, National Chung Hsing University, Taichung, Taiwan

**Keywords:** Lymphocyte cytosolic protein 1 (LCP1), Oral cavity squamous cell carcinoma (OSCC), Interleukin-1β (IL-1β), EGFR signaling pathway, Cancer progression, Proteomics

## Abstract

Oral cancer is the sixth leading cause of cancer-related mortality in Taiwan, with over 90% of cases being oral cavity squamous cell carcinomas (OSCCs). The high mortality rate of OSCC is largely attributed to metastasis and locoregional relapse, underscoring the need to identify key drivers of tumor progression. To uncover proteins involved in OSCC relapse, we conducted an iTRAQ-based proteomic profiling of OSCC tissues from 6 patients with primary tumors and 4 patients with relapsed tumors. Lymphocyte cytosolic protein 1 (LCP1) emerged as a candidate associated with OSCC progression, further supported by transcriptomic analysis from The Cancer Genome Atlas (TCGA). LCP1 showed a 2.4-fold upregulation in relapsed tumors and correlated with poor patient survival. Functional assays revealed that LCP1 expression promoted tumor growth in vivo and enhances proliferation, migration, invasion, and cisplatin resistance in vitro across four OSCC cell lines. Mechanistically, LCP1 expression and phosphorylation were induced by EGF via the EGFR/PI3K/AKT and EGFR/ERK signaling pathways. Additionally, LCP1 activated the JAK2/STAT3 axis to upregulate pro-interleukin-1β (IL-1β) expression and IL-1β secretion, thereby amplifying OSCC cell aggressiveness. In summary, this study provides novel insights into the oncogenic role of LCP1 in OSCC, linking EGFR-mediated signals and IL-1β production, and identifies LCP1 as a promising target for therapeutic intervention.

**Supplementary Information:**

The online version contains supplementary material available at 10.1186/s12935-025-03970-0.

## Introduction

Oral cancer, a subset of head and neck squamous cell carcinoma (HNSCC), is among the most common malignancies worldwide [1]. Over 90% of oral cancers are oral cavity squamous cell carcinoma (OSCC), which are strongly associated with lifestyle factors such as smoking, betel nut chewing, and alcohol consumption. Carcinogens from these behaviors induce genetic and epigenetic alterations that drive malignant transformation [2]. Early-stage OSCC is typically managed with surgical excision followed by cisplatin-based chemotherapy or radiotherapy, whereas late-stage patients often require systematic chemotherapy or targeted therapy. Despite therapeutic advances, OSCC remains fatal in over half of diagnosed cases each year, primarily due to tumor metastasis [3] and recurrence [4]. This underscores the critical need to identify molecular drivers of OSCC progression to inform more effective treatment strategies.

The tumor microenvironment (TME) plays a pivotal role in OSCC development, influencing tumor growth, metastasis, drug resistance, and immune evasion [5, 6]. It also contributes to diagnosis, prognosis, and therapeutic response [7]. To better characterize the OSCC TME, previous studies have profiled the proteome [8, 9] and transcriptome [10, 11] of treatment-naïve OSCC tissues, revealing dysregulated molecules linked to metastasis [10, 11] and metabolic reprogramming [8, 9]. In recurrent OSCC, proteins such as epidermal growth factor receptor (EGFR), matrix metalloproteinases (MMPs) 2 and 9, vascular endothelial growth factor (VEGF), cyclin D1, and p53 have been detected via immunohistochemistry [12, 13]. Kaneko et al.. profiled recurrent oropharyngeal squamous cell carcinoma using Tandem Mass Tag (TMT)-based mass spectrometry [14], yet the proteomic landscape of relapsed OSCC tissues remains unexplored, leaving a critical gap in our understanding of its molecular drivers.

Lymphocyte cytosolic protein 1 (LCP1, also known as L-plastin or plastin-2) is an actin-bundling protein originally identified in leukocytes, where it regulates T cell activation [15] and adhesion [16, 17]. Ectopic expression of LCP1 has been reported in various solid tumors, including colon [18], stomach [19], prostate [20], and triple-negative breast cancers [21], where it promotes cell migration, invasion, and metastasis. In breast cancer, phosphorylation of LCP1 at Ser5 via the PI3K/SGK cascade enhances its oncogenic activity [22]. In OSCC, elevated LCP1 expression has been observed in cancer tissues and is positively correlated with lymphatic metastasis [23]. Moreover, exosomal delivery of LCP1-targeting siRNA suppresses tumor growth in OSCC xenograft mouse models [24]. However, the upstream regulators of LCP1 and its mechanistic role in OSCC progression remain poorly defined.

EGFR is a well-established oncogenic driver in OSCC, frequently overexpressed and associated with poor prognosis [25]. It activates downstream signaling cascades such as PI3K/AKT and ERK, and promotes cell proliferation, survival, and motility [26, 27]. STAT3, another key mediator, is often constitutively activated in OSCC and contributes to immune evasion, inflammation, and metastasis [28, 29]. Both pathways are central to OSCC pathobiology, yet their connection to LCP1 regulation remains unexplored.

In this study, we aimed to identify proteins associated with OSCC progression by comparing proteomic profiles of treatment-naïve and relapsed OSCC tissues using iTRAQ-based mass spectrometry. We identified LCP1 as a candidate driver of recurrence, validated its upregulation via TCGA transcriptomic data, and demonstrated its association with poor survival. Functional assays revealed that LCP1 promotes proliferation, migration, invasion, and cisplatin resistance in OSCC cells. Mechanistically, LCP1 expression and phosphorylation were induced by EGFR signaling through PI3K/AKT and ERK pathways, and further enhanced tumor aggressiveness via JAK2/STAT3-mediated IL-1β production. These findings uncover a novel EGFR–LCP1–STAT3 axis in OSCC and highlight LCP1 as a promising therapeutic target for recurrent disease.

## Materials and methods

### Patient populations and clinical specimens

Tissues for proteome analysis were collected from 6 treatment-naïve OSCC patients (#1-#6) and 4 patients with relapsed OSCC (#5R-#8R). Notably, #5R and #6R correspond to patients #5 and #6 who experienced relapses post-treatment. All oral cancers were resected intraoperatively by an otolaryngologist with adequate surgical margins. Pericancerous tissues were extracted 0.5–1.0 cm from the surgical margins. The tumor margin tissues were sent for fresh frozen-section histopathological analysis. If the margins were determined to be tumor-free, the paired pericancerous tissues could be used as the noncancerous tissues in the proteome analysis. For LCP1 qRT-PCR analysis, tissues were harvested from 224 pretreatment OSCC patients. Patient characteristics and clinical features for both the proteomics and qRT-PCR analyses are detailed in Tables S1 and S9, respectively. All specimens were obtained from Linkou Chang Gung Memorial Hospital (CGMH), Taoyuan, Taiwan, between 2015 and 2020. This study was approved by the Institutional Review Board (IRB) of Linkou CGMH and conducted in accordance with the Declaration of Helsinki (Protocol No. 201800700B0 and 102-5685A3). All participants provided informed consent prior to sample collection. Biopsy-confirmed OSCC cases were diagnosed through oral mucosal screening, and patients underwent routine check-ups following standard protocols.

## Extraction and tryptic digestion of tissue proteins for iTRAQ labeling

Tissue pieces from cancerous and adjacent noncancerous areas were homogenized and digested with trypsin as delineated in Supplemental Materials and Methods. To quantitatively profile tissue proteomes, the resulting tryptic peptides were labeled with iTRAQ reagents (AB Sciex, Foster City, CA, USA). Peptides from cancerous and adjacent noncancerous tissues of pretreatment patients (#1-#6) were labeled with iTRAQ 115 and 114 tags, respectively. Peptides from relapsed cancerous and noncancerous tissues of patients (#5R, #6R, and #8R) were labeled with iTRAQ 117 and 116 tags, respectively, while the cancerous and noncancerous samples form relapsed patient #7R were labeled with iTRAQ 115 and 114 tags, respectively. For the patient #1-#6, the iTRAQ-tagged samples from the same patients were combined, while the iTRAQ-tagged peptides of #7R and #8R were mixed. The combined iTRAQ-tagged peptides were desalted as depicted in Supplemental Materials and Methods.

## Peptide fractionation and mass spectrometry (MS) analysis

The iTRAQ-labeled peptides were separated using online 2-dimensional liquid chromatography (LC; UltiMate™ 3000 RSLCnano System, Thermo Fisher Scientific, San Jose, CA, USA) and analyzed as illustrated in Supplemental Materials and Methods [30]. The LC system was coupled to an LTQ-Orbitrap Elite mass spectrometer (Thermo Fisher Scientific) which was operated with Xcalibur software (version 2.2 SP1.48; Thermo Fisher Scientific). Methods for acquisition of MS and MS/MS data were described in Supplemental Materials and Methods.

## Protein database searching and quantification

Protein database searching and iTRAQ-based quantification were conducted using Proteome Discoverer software (version 1.4.1.14; Thermo Fisher Scientific). The MS/MS spectra were searched against the Swiss-Prot human sequence database (released in March 2023, specific to *Homo sapiens*, containing 20,426 entries) using the Mascot search engine (version 2.2.0; Matrix Science, London, UK). Protein search criteria were described in Supplemental Materials and Methods.

For iTRAQ-based protein quantification, quantitative data were exported from Proteome Discoverer into Excel. Proteins with more than 2 quantifiable spectra were considered quantifiable. The iTRAQ ratios were transformed to a log_2_ scale and manually normalized so that the log_2_ protein ratios displayed a median value of zero. Proteins with log_2_ ratios exceeding the mean plus one standard deviation (SD) were considered overexpressed, while those with log_2_ ratios below the mean minus one SD were considered underexpressed.

## Bioinformatics analysis

Functional enrichment analysis of dysregulated proteins in OSCC tissues was conducted using GO term and biological pathway analyses via STRING (version 11.5). Data visualization and graphing of biological processes and Reactome pathways were performed using SRplot (http://www.bioinformatics.com.cn/SRplot) [31].

For transcriptome analysis of oral cancer tissues, RNA sequencing (RNA-Seq) data from 310 OSCC patients were obtained from the TCGA database through cBioPortal (https://www.cbioportal.org/). Gene expression was calculated using RSEM and batch normalized from Illumina HiSeq_RNASeqV2 data. Survival analysis was conducted based on Z-scores of mRNA expression in OSCC patients. Gene expression correlations between LCP1 and other genes of interest were determined using RNA-Seq data from Taiwanese OSCC patients [32].

### Cell culture

OSCC cell lines KOSC3 (Cat. No. JCRB0127; Japanese Collection of Research Bioresources Cell Bank, Osaka, Japan), OECM-1 (Cat. No. SCC180; Merck, Taipei, Taiwan), SAS (Cat. No. ABL-TC0611; AcceGen Biotechnology, Fairfield, NJ, USA), and SCC25 (Cat. No. 60516; Bioresource Collection and Research Centre, Hsinchu, Taiwan) were cultured in medium supplemented with 10% fetal bovine serum (FBS; Gibco, Waltham, MA, USA) and 1% penicillin/streptomycin solution (P/S; Gibco). KOSC3 and OECM-1 cells were maintained in Roswell Park Memorial Institute 1640 medium (RPMI-1640; Gibco), while SAS cells were grown in Dulbecco’s modified Eagle’s medium (DMEM; Gibco) and DMEM/F-12 medium (Gibco) containing 0.04% hydrocortisone (Sigma-Aldrich) was used for SCC25 cells. All cell lines were incubated at 37 °C with 5% CO_2_.

For mechanistic studies, epidermal growth factor (EGF; Cat. No. 324831; Merck) was used to activate EGF receptor (EGFR) signaling, while cetuximab (Cat. No. MA5-47859; Thermo Fisher Scientific), a monoclonal EGFR antibody, was used as an EGFR inhibitor. GSK1059615 (Cat. No. 11569; Cayman Chemical, Ann Arbor, Michigan, USA), U0126 (Cat. No. 70970; Cayman Chemical), and ruxolitinib (Cat. No. 11609; Cayman Chemical) were utilized to inhibit PI3K, ERK1/2 and JAK1/2, respectively. Cisplatin (Cat. No. p4394; Sigma-Aldrich) was used to suppress OSCC cell growth.

## Transfections

LCP1 expression in KOSC3 and SCC25 cells was inhibited using LCP1 smart pool siRNA (Cat. No. L-011716-00-0005; Dharmacon, Lafayette, CO, USA) transfected with Lipofectamine RNAiMAX (Cat. No. 13778150; Invitrogen, Grand Island, NY, USA). LCP1 smart pool siRNA reagent comprises four individual siRNAs (5’-GGAAGAAUCAAGCGAAGUU-3’, 5’-GAGCGGACAUUUAGGAACU-3’, 5’-GAGGAUCAGUGUCCGAUGA-3’, and 5’-GUACAAGUCUGCCUGUUCU-3’). ON-TARGETplus Non-targeting Control siRNA (Cat. No. D001810-10-20; Dharmacon) was used as the control, containing four siRNAs (5’-UGGUUUACAUGUCGACUAA-3’, 5’-UGGUUUACAUGUUGUGUGA-3’, 5’-UGGUUUACAUGUUUUCUGA-3’, and 5’-UGGUUUACAUGUUUUCCUA-3’). For ectopic LCP1 overexpression, the LCP1-coding sequence was inserted into the pcDNA3.1 C plasmid, and transfection into OECM-1 and SAS cells was performed using TransIT-X2 Transfection Reagent (Cat. No. MR-MIR6000; Mirus Bio, Madison, WI, USA).

To establish stable LCP1-overexpressing cells, lentivirus was produced by co-transfection of pQCXIP-LCP1 and VSV-G into GP2-293T cells. To generate stable LCP1-knockdown cells, lentivirus was produced by co-transfection of shLCP1 (TRCN0000429857), pCMV8.91, and pMD.G plasmids into 293 T cells. OECM-1 and KOSC3 cells were infected with the lentiviruses for LCP1 overexpression and knockdown, respectively. After infection, the cells were selected by puromycin (Cat. No. 101-58-58-2; Cyrusbioscience, Taiwan).

## MTT cell proliferation assay

Growth of OSCC cells (5 × 10^3^ cells/well for KOSC3 and 10^4^ cells/well for OECM-1, SAS, and SCC25) was monitored with MTT (Cat. No. M6494; Invitrogen) assays as described in Supplemental Materials and Methods.

### Cell migration, invasion, and wound healing assays

For wound healing assays, KOSC3 (48-h post transfection; 1.8 × 10^4^ cells/well) and SAS (24-h post transfection; 3.2 × 10^4^ cells/well) cells were seeded into Culture-Insert 4 Well (Cat. No. 80469; ibidi LLC, Verona, WI, USA). The culture-insert was removed at the indicated time points, and the gap areas were quantified using Image J software. For transwell migration and invasion assays, OSCC cells (5 × 10^4^ cells/chamber for OECM-1 and 1.5 × 10^5^ cells/chamber for KOSC3, SAS, and SCC25) were seeded into Transwell Polyester Membrane Inserts (Cat. No. 3464; Corning, Taipei, Taiwan). Invasion and migration assays were performed with and without Matrigel (Cat. No. 354234; Corning)-coated inserts as described in Supplemental Materials and Methods.

### RNA extraction and quantitative real-time PCR (qRT-PCR)

Total RNA was extracted using the TOOLSmart RNA Extractor (Cat. No. DPT-BD24; Biotools, New Taipei City, Taiwan). cDNA was synthesized with the TOOLS@ Quant II Fast RT Kit (Cat. No. KRT-BA06-2; Biotools). qRT-PCR was performed using the TOOLS@ SYBR Green qPCR Mix (Cat. No. FPT-BB05; Biotools). Primer sequences for qRT-PCR are provided in TableS2.

### Extraction of cellular proteins for Western blot

The protein of interest was detected with immunoblotting as detailed in Supplemental Materials and Methods. The primary antibodies used included rabbit anti-LCP1 (1:3000 dilution; Cat. No. 13025-1-AP; Proteintech, Chicago, IL, USA), phospho-LCP1 (Ser5) (1:1000 dilution; Cat. No. 12455-1; Signalway Antibody, Greenbelt, Maryland, USA), rabbit anti-AKT (1:1000 dilution; Cat. No. 9272), phospho-AKT (Ser473) (1:1000 dilution; Cat. No. 9271), rabbit anti- ERK1/2 (1:1000 dilution; Cat. No. 4695), phospho-ERK1/2 (Thr202/Tyr204) (1:1000 dilution; Cat. No. 9101), mouse anti-STAT3 (1:1000 dilution; Cat. No. 9139), phospho-STAT3 (Tyr705) (1:1000 dilution; Cat. No. 9145), mouse anti-IL-1β (1:500 dilution; Cat. No. 12242; all from Cell Signaling, Danvers, MA, USA), mouse anti-GAPDH (1:5000 dilution; Cat. No. sc-32233; Santa Cruz Biotechnology, Santa Cruz, CA, USA), and mouse anti-β-actin (1:5000 dilution; Cat. No. MAB8929; R&D Systems, Minneapolis, MN, USA). Protein bands were quantified using the TotalLab 1D quantitative software (version 14.1; TotalLab, UK). Numbers below each band indicate fold changes relate to the control group.

### Mouse xenograft model

To establish OECM-1 cell line-derived xenografts, 50 µL of an OECM-1 cell suspension (10^7^ cells) was mixed with 50 µL of Matrigel (11.4 mg/mL; Cat. No. 354234; Corning) and then injected subcutaneously into rear flank of NOD.Cg-Prkdc^scid^ Il2rg^tm1Wjl^/SzJ (NSG) mice (obtained from The Jackson Laboratory) with a 26-gauge needle. Tumor formation and growth curves were monitored by Vernier caliper at indicated time-points. Tumor volumes (mm^3^) were calculated with formula: length × width^2^ × 0.5. All animal experiments were approved by Laboratory Animal Center, Chang Gung University (CGU111-082).

### Culture of KOSC3 cells with conditioned media from OECM-1 cells

OECM-1 cells (5 × 10^6^) with and without stable LCP1 overexpression were culture in 15-cm dish for 48 h. The OECM-1 cells were washed three times with PBS and maintained in 15 mL of serum-free RPMI. After 48 h, the culture medium was collected and centrifuged to remove intact cells, and the supernatant after centrifugation was used as the conditioned medium. For MTT assay, KOSC3 cells (10^4^ cells/well) in which LCP1 is stably knocked down were cultured in a 48-well plate with complete medium. After 24 h, the KOSC3 cells were washed with PBS and maintained in 1 mL of OECM-1 conditioned medium. For transwell assay, LCP1-knockdown KOSC3 cells (1.5 × 10^5^ cells) were cultured in a transwell upper chamber with 200 µL of OECM-1 conditioned medium.

### Statistical analysis

Kaplan-Meier plots were stratified by the median LCP1 expression, with *p*-values obtained using the Gehan-Breslow-Wilcoxon test. Differences in *LCP1* gene expression between noncancerous and tumor tissues in OSCC patients were assessed with paired *t*-tests, while differences between disease-free and recurrent groups were evaluated using unpaired *t*-tests. For qRT-PCR, proliferation, migration, invasion, and wound healing assays, between-group comparisons were performed using Student’s *t*-tests or one-way ANOVA. Correlations between *LCP1* and genes of interest were determined using Pearson’s correlation coefficient. Each experiment was repeated three times, and consistent trends across the replicates were required for conclusions. Data presented are from one of the three replicates. Statistical tests were two-sided, with a *p*-value < 0.05 considered statistically significant. All analyses were conducted using Prism software (version 9.0; GraphPad Software, La Jolla, CA, USA).

## Results

### Proteomics analysis of OSCC tissues from treatment-naïve and recurrent patients

To identify proteins associated with OSCC, we profiled the proteomes of tumor and adjacent noncancerous tissues from patients with pretreatment OSCC (#1-#6) and recurrent OSCC (#5R-#8R). Notably, patients #5R and #6R were the same as patients #5 and #6, who experienced a relapse more than three years after their initial treatment (Table S1). For each patient, adjacent noncancerous epithelial (N) and tumor (T) tissues were comparatively analyzed using iTRAQ labeling combined with 2D-LC-MS/MS.

As detailed in Table S3, an average of 4,661 proteins were quantified in the pretreatment OSCC tissues, with individual counts of 4,153, 4,558, 4,991, 5,199, 4,494, and 4,570 proteins for patients #1-#6, respectively. In the recurrent OSCC tissues, an average of 4,674 proteins were quantified, with individual counts of 4,541, 4,570, 4,776, and 4,808 proteins for patients #5R-#8R, respectively. Detailed information on peptide identification and protein quantification in OSCC tissues is provided in **T**ables S4 and S5.

To identify differentially expressed proteins in OSCC tissues, the means and standard deviations (SDs) of the T/N ratios for all proteins were calculated for each patient (Table S3). Proteins with T/N ratios exceeding the mean plus one SD were classified as overexpressed in cancer tissue, while those with ratios below the mean minus one SD were considered underexpressed. In the pretreatment OSCC tissues, we identified an average of 486 upregulated proteins (442, 448, 464, 539, 505, and 518 proteins in patients #1-#6, respectively) and 544 downregulated proteins (514, 479, 534, 531, 522, and 681 proteins in patients #1-#6, respectively). In the recurrent OSCC tissues, we identified an average of 449 upregulated proteins (347, 492, 497, and 459 proteins in patients #5R-#8R, respectively) and 537 downregulated proteins (445, 557, 585, and 560 proteins in patients #5R-#8R, respectively).

### Dysregulated proteins and biological pathways in treatment-naïve and recurrent OSCC tissues

**Fig. 1:**
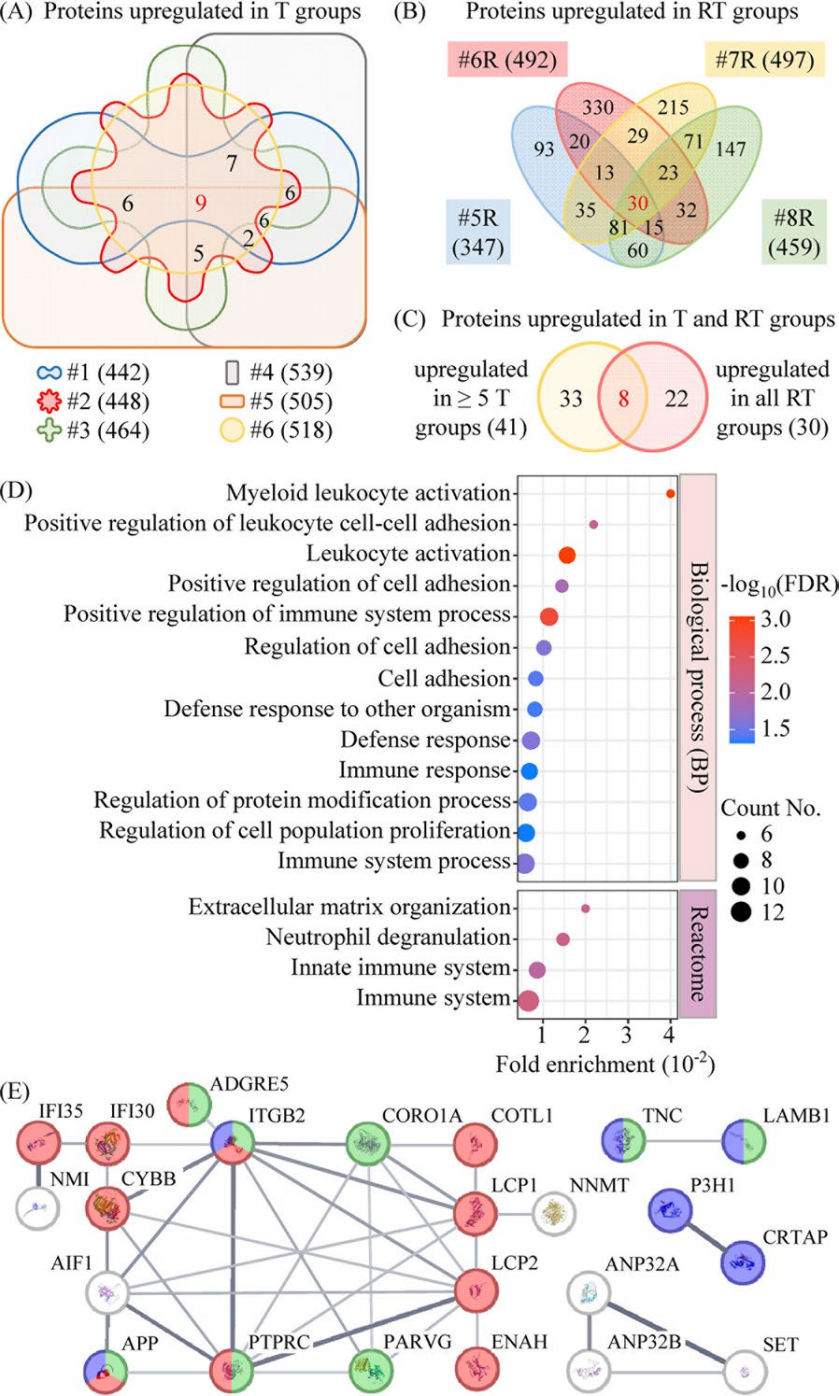
Proteome profiling of tumor tissues collected from patients with primary and relapsed OSCC. Tumor tissues and their case-matched adjacent noncancerous tissues were collected from 6 patients with primary OSCCs (#1-#6) and 4 patients with relapsed OSCCs (#5R-#8R). The proteome of primary tumors (T), adjacent noncancerous tissues of T (N), relapsed tumors (RT), and adjacent noncancerous tissues of RT (RN) was analyzed using iTRAQ-based mass spectrometry. Protein ratios of T/N and RT/RN were calculated. The mean and standard deviation (SD) of the ratios for all proteins in each comparison were determined, with proteins having ratios above the mean + SD considered overexpressed. (**A**) Venn diagram illustrating the overlap of upregulated proteins in the T groups. Red and black numbers indicate the number of proteins elevated in all T groups and in 5 out of 6 T groups, respectively. (**B**) Venn diagram showing overlaps of upregulated proteins in the RT groups. (**C**) Venn diagram displaying overlap between upregulated proteins in the T and RT groups, with total identified proteins listed in brackets. (**D**) Functional annotation of the 30 proteins upregulated in the RT groups was performed using STRING v12.0 software, and the results of biological process and Reactome pathway analyses were processed with SRplot online software. (**E**) A protein-protein interaction network of the 30 upregulated proteins was constructed, showing 38 interaction links (solid lines). Modules within the network highlight interactions of proteins involved in immune system pathways (red nodes), cell adhesion (green nodes), and ECM organization (blue nodes)

To minimize the impact of individual variations, we focused on 207 proteins consistently dysregulated in at least 5 pretreatment OSCC tissues, comprising 41 overexpressed and 166 underexpressed proteins (Figs. 1A and S1A). In recurrent OSCC tissues, 30 proteins were consistently upregulated, and 169 were downregulated (Figs. 1B and S1B). Detailed information on these dysregulated proteins is provided in Table S6.

To investigate OSCC progression and recurrence, we performed an overlap analysis of differentially expressed proteins in pretreatment and recurrent OSCC tissues. As shown in Fig. 1C, among the 63 proteins upregulated in either primary or recurrent cancer tissues, 8 (12.7%) were common to both, while 110 (48.9%) of the 225 downregulated proteins were shared (Fig. S1C). These findings suggest that proteins involved in OSCC progression and recurrence may differ from those driving initial OSCC development, particularly those upregulated in OSCC tissues.

To explore the biological processes and pathways associated with these dysregulated proteins, we performed functional annotation using the STRING database. The 41 proteins upregulated in pretreatment OSCCs were significantly associated with pathways related to extracellular matrix (ECM) organization, ECM-receptor interaction, collagen formation, and interferon signaling. In contrast, the 30 proteins upregulated in recurrent OSCCs were primarily involved in ECM organization and immune system regulation **(**Fig. 1D and Table S7). Enrichment analysis further confirmed the strong association of these proteins with immune system processes and cell adhesion (Fig. 1D).

Protein-protein interaction (PPI) networks for the 30 upregulated proteins in recurrent OSCCs were predicted using the STRING database, revealing 38 interaction links. These interactions highlighted modules related to immune system processes, cell adhesion, and ECM organization (Fig. 1E), consistent with the biological process and pathway analyses.

For the proteins downregulated in either pretreatment or recurrent OSCC tissues, pathways related to complement and coagulation cascades, cholesterol/lipid metabolism, and ECM-receptor interaction were identified. Notably, the biological processes and pathways associated with the 166 downregulated proteins in primary OSCCs were similar to those revealed by the 169 downregulated proteins in recurrent OSCCs (Table S7), reflecting the high overlap observed between these two groups.

### Evaluation of LCP1 as an OSCC progression-associated protein

Enrichment analysis revealed that proteins upregulated in recurrent OSCC tissues were strongly associated with immune responses and extracellular matrix (ECM) organization within the tumor microenvironment (Fig. 1D). To pinpoint proteins linked to OSCC progression, we concentrated on the 30 proteins with elevated levels in recurrent OSCC tissues (Table S6). Using RNA-Seq data from the TCGA database via the cBioPortal, we assessed whether the expression of these 30 genes correlated with patient survival in OSCC. As shown in Table S8, higher expression of 9 genes—*ADGRE5*, *AIF1*, *ARHGAP45*, *CORO1A*, *COTL1*, *CRTAP*, *IFI30*, *LCP1*, and *NNMT*—was associated with poorer disease-free, disease-specific, or progression-free survival in OSCC patients.

To further validate the connection between these 9 genes and OSCC progression, their expression was analyzed in advanced OSCC tissues. As shown in Fig. S1D, three genes—*ADGRE5*, *COTL1*, and *LCP1*—were significantly upregulated in OSCCs with lymph node metastasis compared to those without lymphatic metastasis. Additionally, the expression of *AIF1*, *CORO1A*, *CRTAP*, and *LCP1* was higher in primary tumors of OSCC patients who experienced recurrence after treatment than in those who remained disease-free (Fig. S1E).

**Fig. 2:**
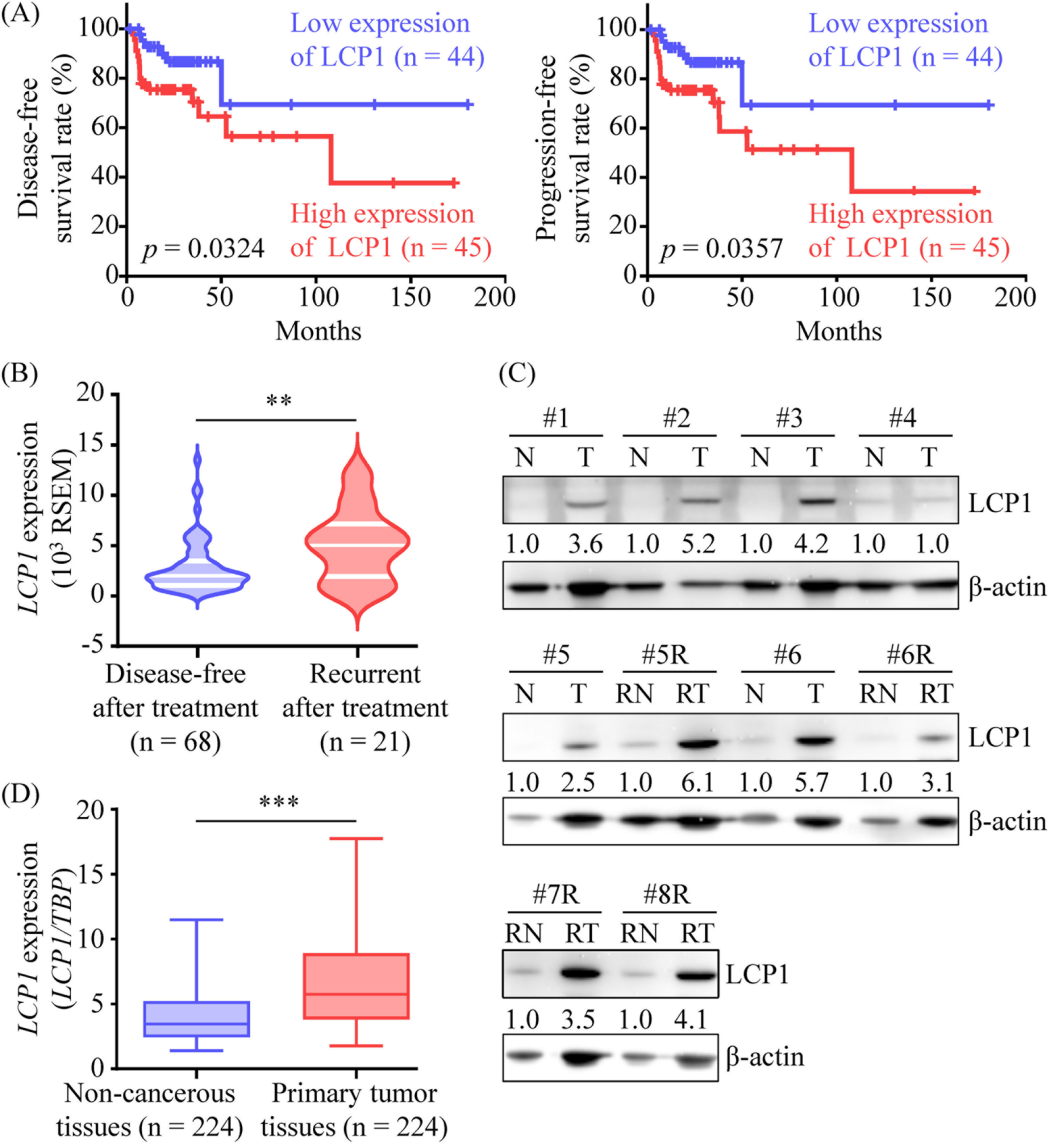
LCP1 upregulation in tumor tissues was associated with poor survival of OSCC patients. The expression of *LCP1* gene in OSCC tissues was analyzed using RNA-Seq data from the cBioPortal website. (**A**) Kaplan-Meier plots display disease-free (left panel) and progression-free (right panel) survival of 89 OSCC patients, stratified by the median *LCP1* expression level. *P*-values were calculated using the Gehan-Breslow-Wilcoxon test. (**B**) *LCP1* gene expression in primary tumors was compared between OSCC patients who relapsed after treatment (*n* = 21) and those who remained disease-free post-treatment (*n* = 68). The data are presented in a violin plot, with quartiles indicated by a white line. Differences between the groups were determined using parametric unpaired *t*-tests (**p < 0.01). (**C**) Tumor and matched adjacent noncancerous tissues were collected from 6 patients with primary OSCCs (#1-#6) and 4 patients with relapsed OSCCs (#5R-#8R). LCP1 protein levels in primary tumors (T), adjacent noncancerous tissues of T (N), relapsed tumors (RT), and adjacent noncancerous tissues of RT (RN) were detected by Western blot. LCP1 levels in the cancer group were presented as fold changes relative to the noncancerous group. (**D**) *LCP1* gene expression was compared between primary tumor tissues and corresponding adjacent noncancerous epithelia in Taiwanese OSCC patients (*n* = 224) using qRT-PCR, with *TBP* Gene expression serving as the internal control. The results are shown as box-and-whisker plots, where the whiskers, boxes, and horizontal lines represent the middle 90%, upper and lower quartiles, and medians of *LCP1* expression, respectively. Differences between groups were determined using non-parametric paired *t*-tests (***p < 0.001)

Among these, LCP1 was selected for further investigation due to its strong correlation with poorer patient survival (Fig. 2A and Table S8) and its association with advanced OSCC (Figs. 2B, S1D, and S1E). LCP1 protein levels in OSCC tissues were first evaluated using the samples from the iTRAQ-based MS analysis. As shown in Fig. 2C, LCP1 levels were at least 3-fold higher in 4 primary cancer tissues (#1, #2, #3, and #6) compared to their adjacent noncancerous counterparts, and its expression was elevated in all recurrent cancer tissues relative to the corresponding noncancerous epithelia.

We further assessed *LCP1* Gene expression in pretreatment cancer tissues from 224 OSCC patients using qRT-PCR. *LCP1* was significantly upregulated in OSCC tissues compared to paired noncancerous epithelia (2.1-fold increase; *p* < 0.01; Fig. 2D). However, in this case-control study, *LCP1* expression in primary OSCC tissues was not significantly associated with age, sex, lymphatic metastasis, overall stage, cell differentiation, or tumor depth (Table S9).

### LCP1 enhances the proliferation and migration of OSCC cells

**Fig. 3:**
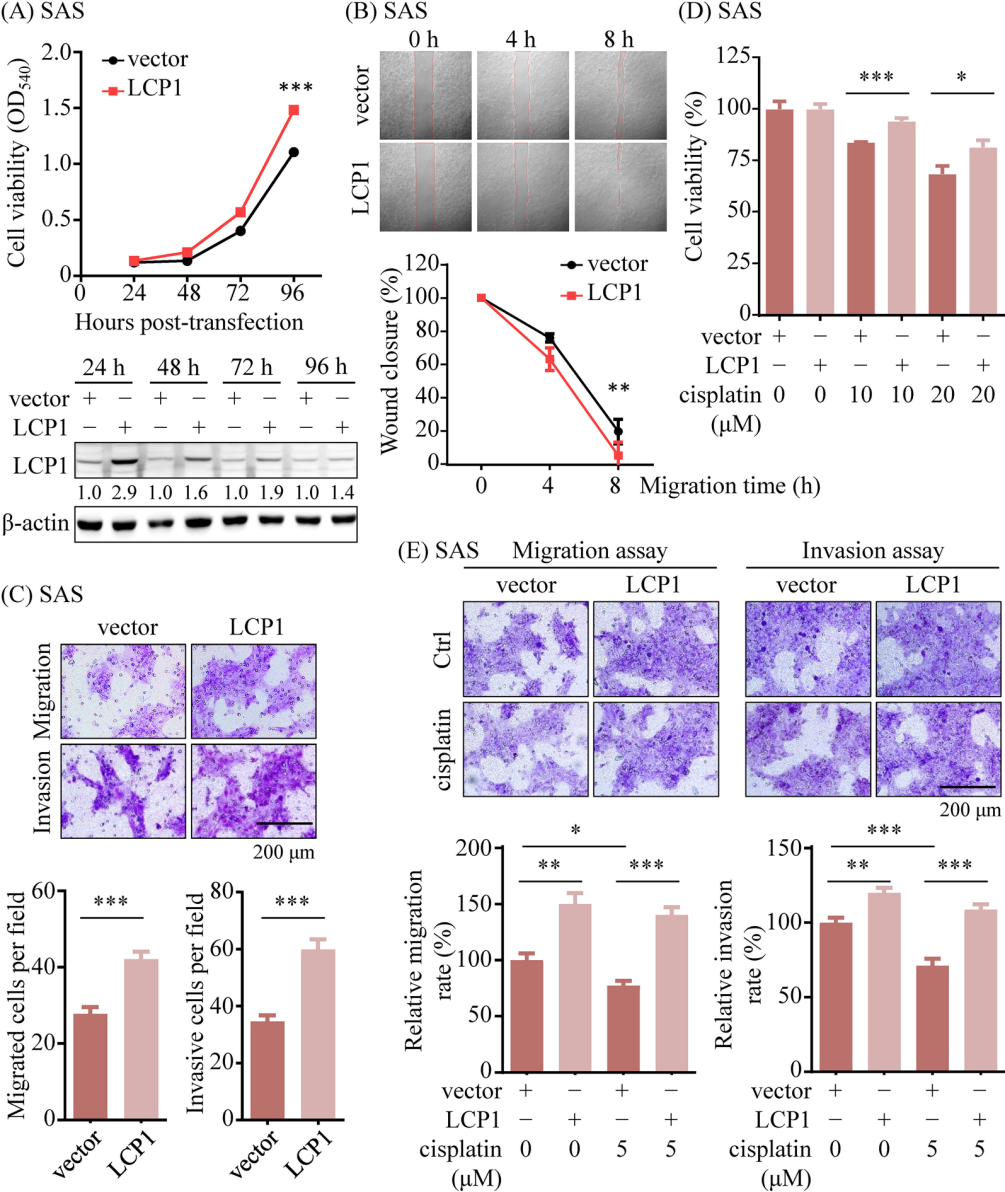
LCP1 overexpression enhanced proliferation, migration, and cisplatin resistance in OSCC cell lines. (**A**) MTT assay for cell proliferation and (**B**) wound healing assays were performed using SAS cells transfected with either a control vector or an LCP1 expression plasmid. (**C**) Transwell migration and Matrigel invasion assays (original magnification, ×400; scale bar: 200 μm) were conducted to assess the effects of LCP1 overexpression. (**D**) The proliferation and (**E**) migration and invasion capabilities of LCP1-overexpressing SAS cells were evaluated with and without cisplatin treatment. Quantification of migration and invasion abilities (**C**, **E**) is presented in bar graphs. Statistical significance was determined using parametric unpaired *t*-tests (**A**-**D**) and one-way ANOVA (E). *p < 0.05, **p < 0.01, ***p < 0.001

 To investigate the role of LCP1 in OSCC progression, we evaluated the impact of LCP1 overexpression on cell growth and motility in the OSCC cell lines, SAS and OECM-1. As shown in Figs. 3A and S2A, overexpression of LCP1 significantly promoted cell viability (1.5-fold increase; *p* < 0.001). Increase of LCP1 levels enhanced wound healing migration of SAS cells (Fig. 3B). Transwell assays further demonstrated that LCP1 overexpression enhanced both the migratory and invasive properties of SAS and OECM-1 cells (Figs. 3C and S2B). Conversely, silencing LCP1 expression markedly suppressed proliferation, migration, and invasion in the OSCC cell lines, KOSC3 and SCC25 (Figs. S3A-S3C, S4A, and S4B).

We also explored the role of LCP1 in cisplatin resistance in OSCC cells. Ectopic expression of LCP1 rendered SAS and OECM-1 cells less sensitive to cisplatin treatment (Figs. 3D and S2C), while knockdown of LCP1 enhanced the cisplatin-induced suppression of proliferation in KOSC3 and SCC25 cells (Figs. S3D and S4C). Moreover, LCP1 overexpression counteracted the inhibitory effects of cisplatin on OSCC cell migration and invasion (Figs. 3E and S2D). In contrast, inhibiting LCP1 expression further amplified the cisplatin-induced suppression of migration and invasion in OSCC cells (Figs. S3E and S4D).

### LCP1 overexpression promotes xenograft tumor growth

**Fig. 4:**
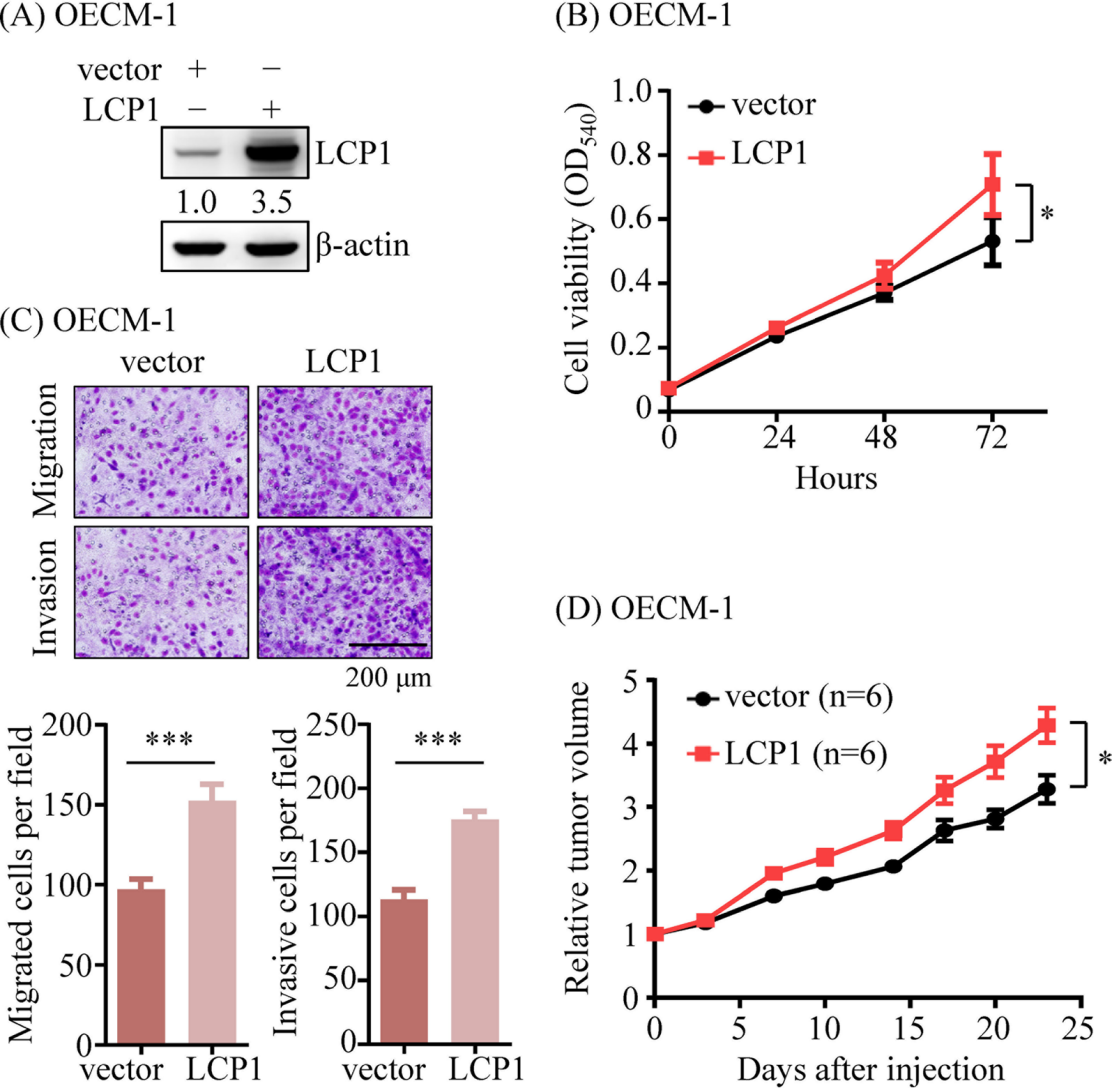
LCP1 promoted the xenograft tumor growth. (**A**) Stable LCP1-overexpressing OECM-1 cells were established with infection of lentivirus, which were produced by co-transfecting pQCXIP-LCP1 and VSV-G into GP2-293T cells. (**B**) MTT assay for cell proliferation was performed using stable LCP1-overexpressing OECM-1 cells and control cells. (**C**) Transwell migration and Matrigel invasion assays (original magnification, ×400; scale bar: 200 μm) were conducted to assess the effects of stable LCP1 overexpression. Quantification of migration and invasion abilities is presented in bar graphs. (**D**) OECM-1 cells (1 × 10^7^) with and without stable overexpression of LCP1 were inoculated subcutaneously into the right flank of NSG mice. Data are represented as the mean ± SEM of relative tumor volumes from six mice. Statistical significance was determined using parametric unpaired *t*-tests. *p < 0.05, **p < 0.01, ***p < 0.001

 To determine the tumorigenic role of LCP1, stable LCP1-overexpressing OECM-1 cells were established (Fig. 4A). Overexpression of LCP1 significantly promoted growth (1.4-fold increase; *p* < 0.05), migration (1.5-fold increase; *p* < 0.001), and invasion (1.6-fold increase; *p* < 0.001) abilities in OECM-1 cells (Fig. 4B and C). The LCP1-overexpressing OECM-1 cells were then assessed in vivo for tumor formation. The results recapitulated those of the cell-based assays, with the LCP1-overexpressing group exhibiting significantly larger tumor volumes compared to the control group (1.3-fold increase; *p* < 0.05; Fig. 4D). These findings collectively illustrate a functional link between LCP1 upregulation and the enhanced progression potential of OSCC.

#### LCP1 and its phosphorylation are upregulated via EGFR signaling pathways in OSCC cells

Having established a functional link between LCP1 and the progressive capabilities of OSCC cells, we next investigated the upstream pathways responsible for the ectopic expression of LCP1 in OSCC. Machado et al.. used computational modeling and immunoblot data to identify the PI3K and ERK pathways as upstream regulators of LCP1 in breast cancer cells [22]. EGFR signaling, a key mediator of the PI3K/AKT and MAPK/ERK pathways, is known to contribute to cisplatin resistance and progression in oral cancer [33, 34]. Given that EGFR expression in OSCC ranges from 40 to 100% [10], we examined how EGFR signaling activation affects LCP1 expression.

**Fig. 5:**
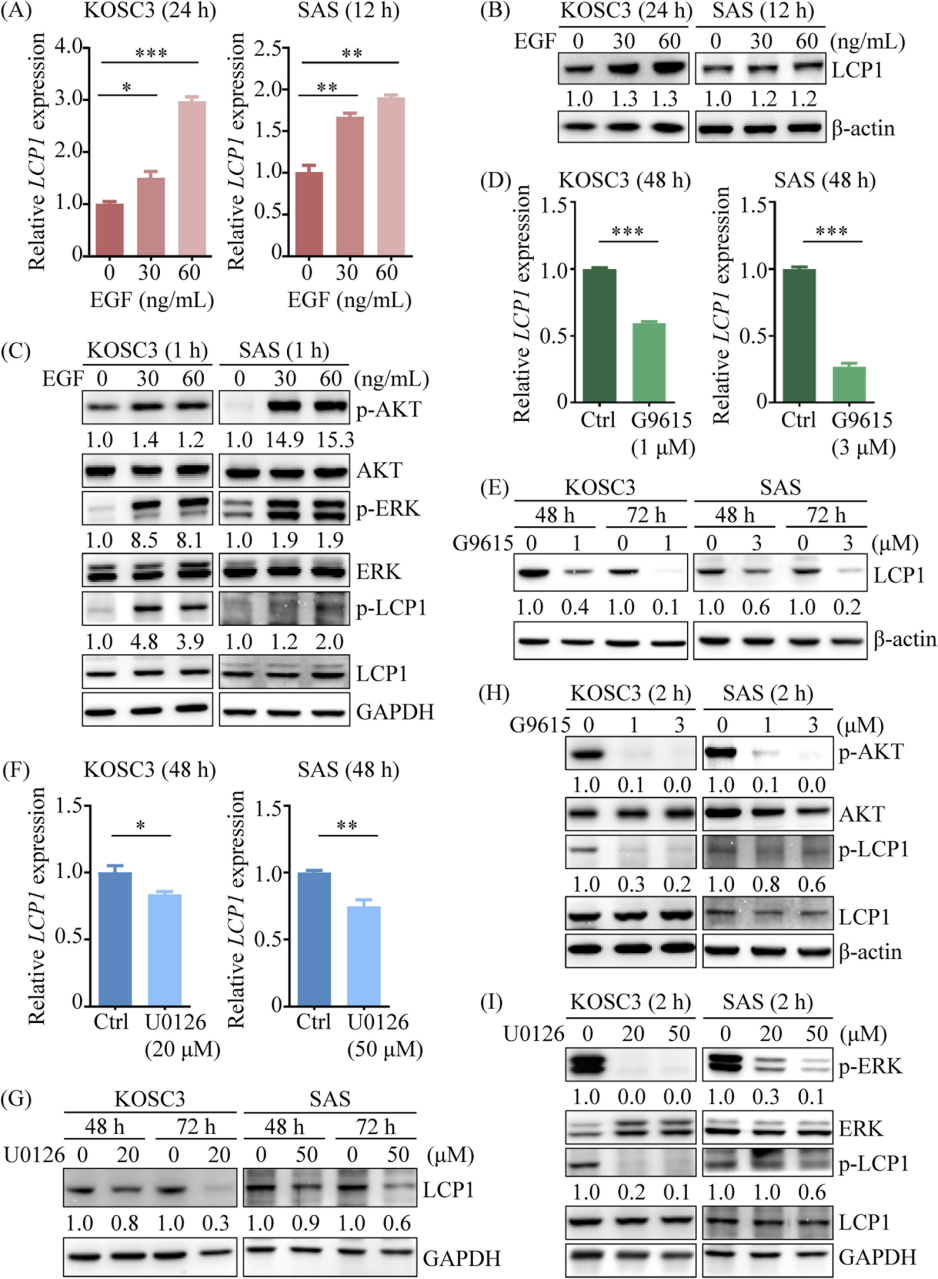
LCP1 expression and Ser5 phosphorylation were upregulated in OSCC cells via EGFR signaling pathway. (**A**, **B**) LCP1 expression levels were analyzed in the KOSC3 and SAS cells treated with EGF for 24 h and 12 h, respectively, using qRT-PCR (**A**) and Western blot (**B**). (**C**) Phosphorylation of AKT (Ser473; p-AKT), ERK1/2 (Thr202/Tyr204; p-ERK), and LCP1 (Ser5; p-LCP1) was assessed in OSCC cells following 1 h of EGF treatment. (**D**-**I**) OSCC cells were treated with the PI3K inhibitor GSK1059615 (G9615; **D**, **E**, **H**) or the ERK inhibitor U0126 (F, G, I). LCP1 expression was measured by qRT-PCR after 48–72 h of inhibitor treatment (**D**, **F**) and by Western blotting (**E**, **G**). Phosphorylation of LCP1 at Ser5 was detected using immunoblotting after 2 h of inhibitor treatment (**H**, **I**). Internal control genes for qRT-PCR analyses in (**A**), (**D**), and (**F**) are *RPN18S*, *ACTN*, and *GAPDH*, respectively. Statistical significance was determined by Student’s *t*-test. *p < 0.05, **p < 0.01, ***p < 0.001

Our qRT-PCR analysis revealed that EGF treatment enhanced *LCP1* expression in OSCC cells in a dose-dependent manner (Fig. 5A). Specifically, EGF stimulation significantly increased LCP1 protein levels in KOSC3 cells, while LCP1 levels were only slightly elevated in SAS cells (Fig. 5B). EGF treatment also resulted in increased phosphorylation of AKT and ERK, as shown by our detection of these phosphorylated proteins (Fig. 5C). To elucidate the relationship between LCP1 expression and the PI3K/AKT or MAPK/ERK pathways, we used GSK1059615 and U0126 to inhibit PI3K and ERK activity, respectively. Inhibition of PI3K (Fig. 5D and E) and ERK (Fig. 5F and G) reduced LCP1 expression in OSCC cells. The effectiveness of these inhibitors was confirmed by assessing the phosphorylation status of AKT (Fig. 5H) and ERK (Fig. 5I).

Phosphorylation of LCP1 on Ser5 enhances its actin-binding activity [35], so both LCP1 expression and its phosphorylation are important for understanding LCP1’s role in OSCC progression. EGF stimulation for one hour did not alter LCP1 expression but did induce Ser5 phosphorylation of LCP1 in OSCC cells (Fig. 5C). Moreover, inhibition of PI3K and ERK reduced LCP1 Ser5 phosphorylation (Fig. 5H and I). These findings suggest that LCP1 expression and phosphorylation are regulated by the EGFR/PI3K/AKT and EGFR/MAPK/ERK signaling pathways in OSCC cells.

**Fig. 6:**
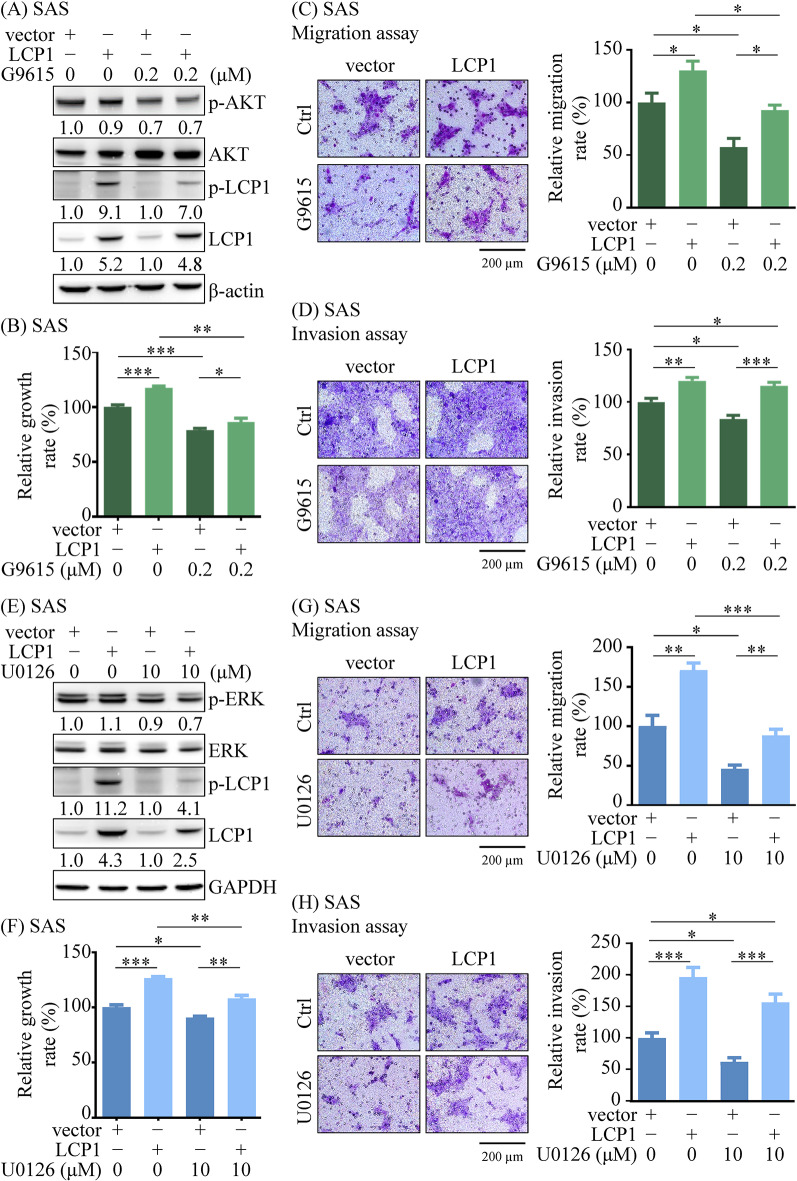
PI3K and ERK inhibitors suppressed LCP1-mediated proliferation and migration in OSCC cell lines. SAS cells were transfected with either a control vector or an LCP1 expression plasmid. (**A**-**D**) After 24 h of transfection, the SAS cells were treated with 0.2 µM PI3K inhibitor GSK1059615 (G9615) for 24 h. Western blot analysis (**A**), MTT cell proliferation assay (**B**), transwell migration assay (**C**; original magnification, ×400; scale bar: 200 μm), and Matrigel invasion assay (**D**; original magnification, ×400; scale bar: 200 μm) were performed. (**E-H**) LCP1-overexpressing SAS cells were treated with 10 µM ERK inhibitor U0126. After 24 h, Western blot analysis (**E**), MTT cell proliferation assay (**F**), transwell migration assay (**G**), and Matrigel invasion assay (H) were conducted. Proliferation, migration, and invasion abilities were quantified and presented in bar graphs. Statistical significance was determined using one-way ANOVA. *p < 0.05, **p < 0.01, ***p < 0.001

Given LCP1’s role in promoting OSCC progression, we further explored the functional link between LCP1’s upstream regulators and OSCC cell progression. Functional assays demonstrated that proliferation, migration, and invasion were significantly reduced in SAS cells treated with 0.2 µM GSK1059615 and 10 µM U0126 compared to control groups (Fig. 6). LCP1 overexpression led to increased Ser5 phosphorylation and counteracted the inhibitory effects of PI3K and ERK inhibitors on cell proliferation, migration, and invasion (Fig. 6). These results indicate that LCP1 is upregulated by the EGFR/PI3K/AKT and EGFR/MAPK/ERK cascades and enhances cell proliferation, migration, and invasion in OSCC cells.

### JAK/STAT3 is a downstream signaling pathway of LCP1 in OSCC cells

**Fig. 7:**
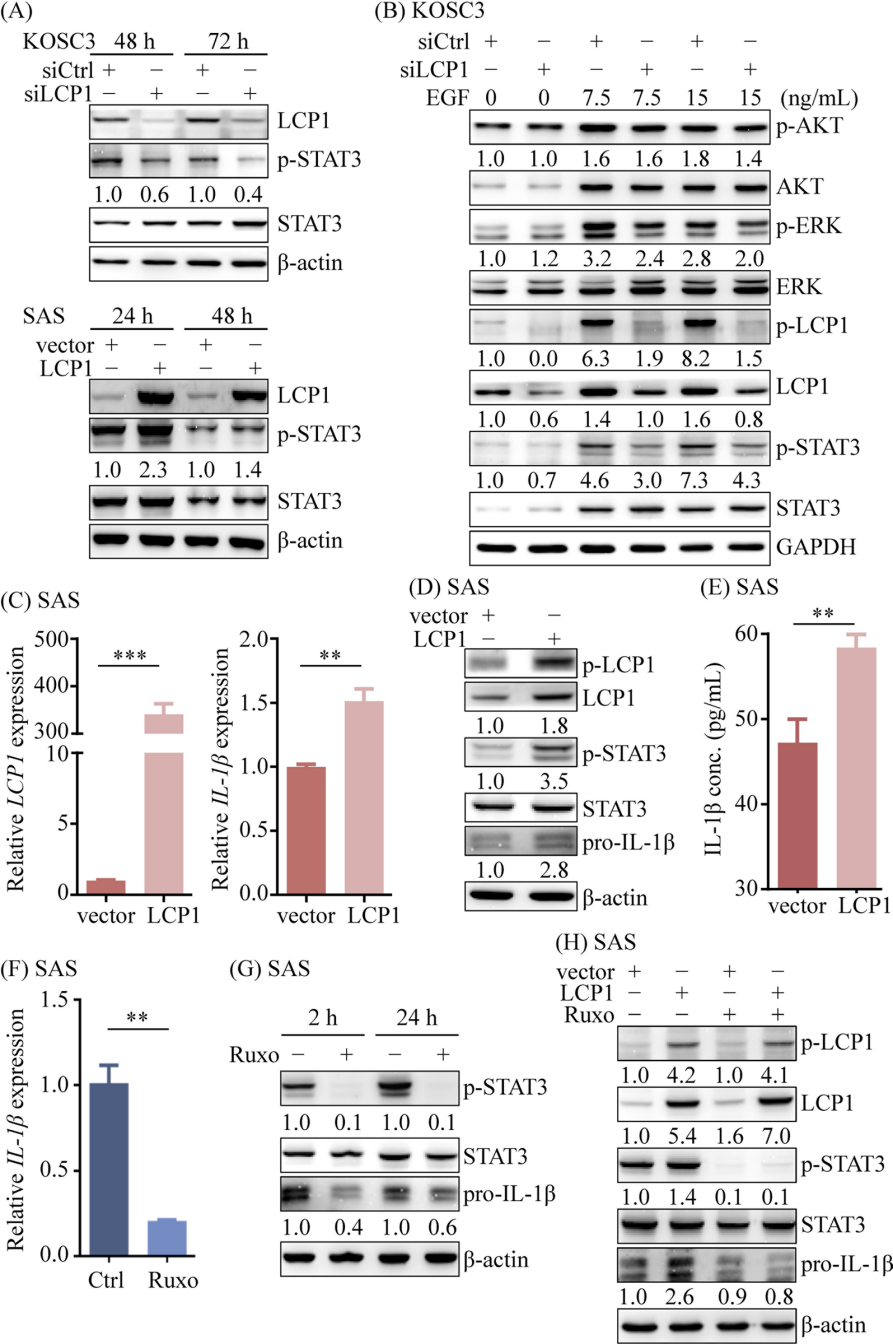
LCP1 upregulated pro-IL-1β expression via the JAK/STAT3 axis in OSCC cells. (**A**) Phosphorylation of STAT3 at Tyr705 was assessed in KOSC3 cells transfected with control siRNA (siCtrl) or LCP1-specific siRNA (siLCP1), and in SAS cells transfected with either a control vector or an LCP1 expression plasmid. (**B**) LCP1-knockdown KOSC3 cells were treated with EGF for 1 h, and proteins of interest were detected using Western blotting with the indicated antibodies. (**C**-**E**) SAS cells were transfected with either a control vector or an LCP1 expression plasmid. After 24 h, LCP1 and pro-IL-1β expression levels were measured using qRT-PCR (**C**) and Western blotting (**D**). IL-1β levels in 48-hour serum-free conditioned media were quantified using a bead-based ELISA (**E**). (**F**, **G**) SAS cells were treated with 10 µM JAK1/2 inhibitor ruxolitinib (Ruxo). After 24 h, expression of pro-IL-1β were determined using qRT-PCR (**F**). Proteins of interest were detected with Western blotting after 2 h and 24 h of ruxolitinib treatment (**G**). (**H**) SAS cells overexpressing LCP1 were treated with 10 µM ruxolitinib for 2 h following 24 h of LCP1 overexpression. Whole-cell lysates were analyzed by immunoblotting with the indicated specific antibodies. Pro-IL-1β levels in experimental groups are presented as fold changes relative to the control group. *ACTN* is used as an internal control gene for the qRT-PCR analyses. Statistical significance was determined by unpaired Student’s *t*-test. *p < 0.05. **p < 0.01. ***p < 0.001

LCP1 has been reported to promote proliferation and metastasis in osteosarcoma cells via the JAK2/STAT3 pathway [36]. Given the critical roles of the JAK2/STAT3 signaling pathway in the proliferation and invasion of OSCC cells [37, 38], we investigated the relationship between LCP1 and JAK2/STAT3 signaling in OSCC tissues. As shown in Fig. S5, there was a significant positive correlation between the gene expression of LCP1 and STAT3 (*r* = 0.45; *p* = 0.0094) in OSCC tissues. Notably, the correlation between LCP1 and JAK2 was even stronger (*r* = 0.67; *p* < 0.0001), suggesting a robust association between LCP1 and the JAK2/STAT3 signaling cascade. Given the upregulation of LCP1 in OSCC tissues, it is likely that the LCP1/JAK2/STAT3 axis is activated in these tissues. As shown in Figs. 7A and S6A, Western blot analysis indicated that the phosphorylation of STAT3 at tyrosine 705, a primary site phosphorylated by activated JAK, was reduced in LCP1-knockdown KOSC3 and SCC25 cells. Conversely, LCP1 overexpression increased STAT3 phosphorylation in SAS and OECM-1 cells. These findings suggest that LCP1 regulates the JAK/STAT3 pathway in OSCC cells.

To further understand the role of EGFR signaling in JAK/STAT3 activation, we assessed the effects of EGFR signaling on JAK/STAT3 in OSCC cells. EGF treatment increased STAT3 phosphorylation, while LCP1 knockdown diminished EGF-induced STAT3 phosphorylation in KOSC3 and SCC25 cells (Fig. 7B and S6B). Collectively, these results demonstrate that the JAK/STAT3 pathway is regulated by EGFR signaling, which acts upstream of LCP1.

#### LCP1 induces IL-1β production via the JAK/STAT3 signaling pathway in OSCC cells

LCP1 has been shown to enhance the NLRP3 inflammasome, which regulates IL-1β production in macrophages [39]. In OSCC, tumor-derived IL-1β can act in a paracrine manner to promote ECM degradation and tumor cell invasion [40, 41]. We therefore investigated whether LCP1 enhances IL-1β production in OSCC cells. As illustrated in Figs. 7C and S6C, ectopic expression of LCP1 significantly increased IL-1β levels in SAS (1.5-fold increase; *p* < 0.01) and OECM-1 (1.5-fold increase; *p* < 0.01) cells. Western blot analysis revealed level of precursor IL-1β (pro-IL-1β) was higher in LCP1-overexpressing SAS and OECM-1 cells compared to controls (Figs. 7D and S6D), while LCP1 knockdown reduced pro-IL-1β expression in SCC25 cells (Figs. S6E and S6F). Additionally, mature IL-1β was elevated in the conditioned media of LCP1-overexpressing SAS cells (Fig. 7E), indicating enhanced secretion.

Given prior evidence that miR-216a suppresses IL-1β production via inhibition of the JAK2/STAT3 pathway in neuronal cells [42], and our observation that LCP1 activates JAK/STAT3 signaling in OSCC cells (Fig. 7A and B, S6A, and S6B), we examined whether IL-1β upregulation is mediated through this axis in OSCC. Treatment with the JAK1/2 inhibitor ruxolitinib reduced IL-1β levels in OSCC cells compared to controls (Fig. 7F and G, and S6G). Moreover, ruxolitinib partially reversed the increase in IL-1β induced by LCP1 overexpression in SAS and OECM-1 cells (Figs. 7H and S6H).

We also assessed NF-κB activation, a known regulator of pro-IL-1β transcription [43]. NF-κB phosphorylation levels were not significantly altered in LCP1-overexpressing SAS cells or LCP1-knockdown KOSC3 cells (Figs. S7A and S7B). Furthermore, ruxolitinib treatment did not affect NF-κB phosphorylation (Fig.S7C), suggesting that IL-1β production in OSCC cells is regulated primarily through the LCP1/JAK/STAT3 axis rather than the NF-κB pathway.

#### IL-1β promotes OSCC cell growth and migration

**Fig. 8:**
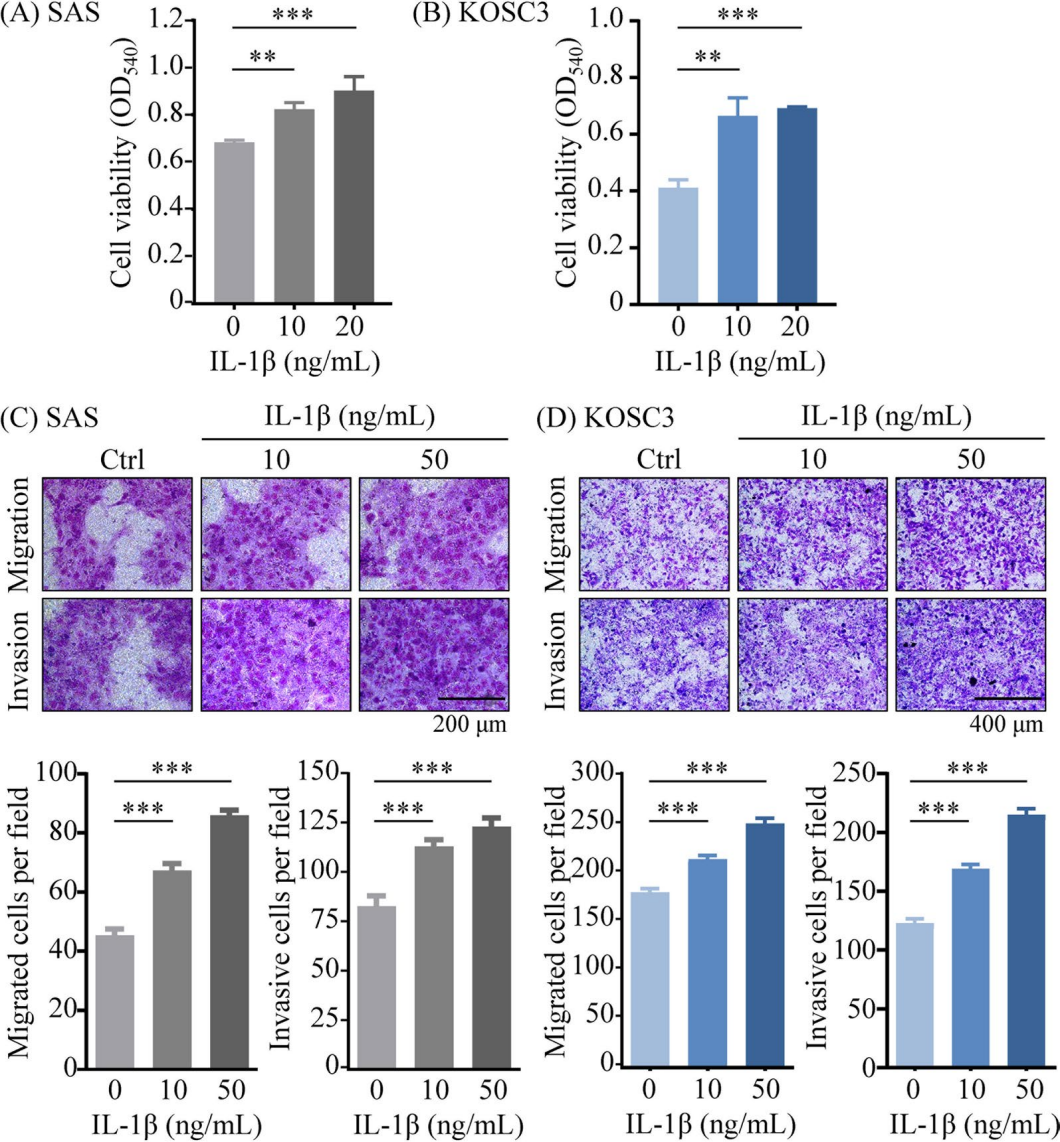
Recombinant IL-1β enhanced growth and migration in OSCC cells. (**A**, **B**) OSCC cells were cultured in serum-free media for 24 h. The growth of SAS (**A**) and KOSC3 (**B**) cells was then assessed after 48 h of IL-1β stimulation using the MTT assay. (**C**, **D**) The migratory and invasive abilities of OSCC cells were evaluated using transwell migration and invasion assays. SAS cells in the upper chamber were treated with IL-1β for 24 h (**C**; original magnification, ×400; scale bar: 200 μm). KOSC3 cells in the upper chamber were treated with IL-1β for 24 h in the migration assay and 18 h in the invasion assay (**D**; original magnification, ×200; scale bar: 400 μm). Proliferation, migration and invasion abilities were quantified and presented in the bar graph. Statistical significance was determined by unpaired Student’s *t*-test. *p < 0.05, **p < 0.01, ***p < 0.001

Given LCP1’s role in promoting OSCC progression, we assessed the effects of IL-1β treatment on OSCC cell growth and motility. IL-1β treatment enhanced OSCC cell proliferation in a dose-dependent manner (Fig. 8A and B) and significantly increased migration and invasion (Fig. 8C and D), suggesting involvement of IL-1β in LCP1-mediated functions in OSCC cells.

**Fig. 9:**
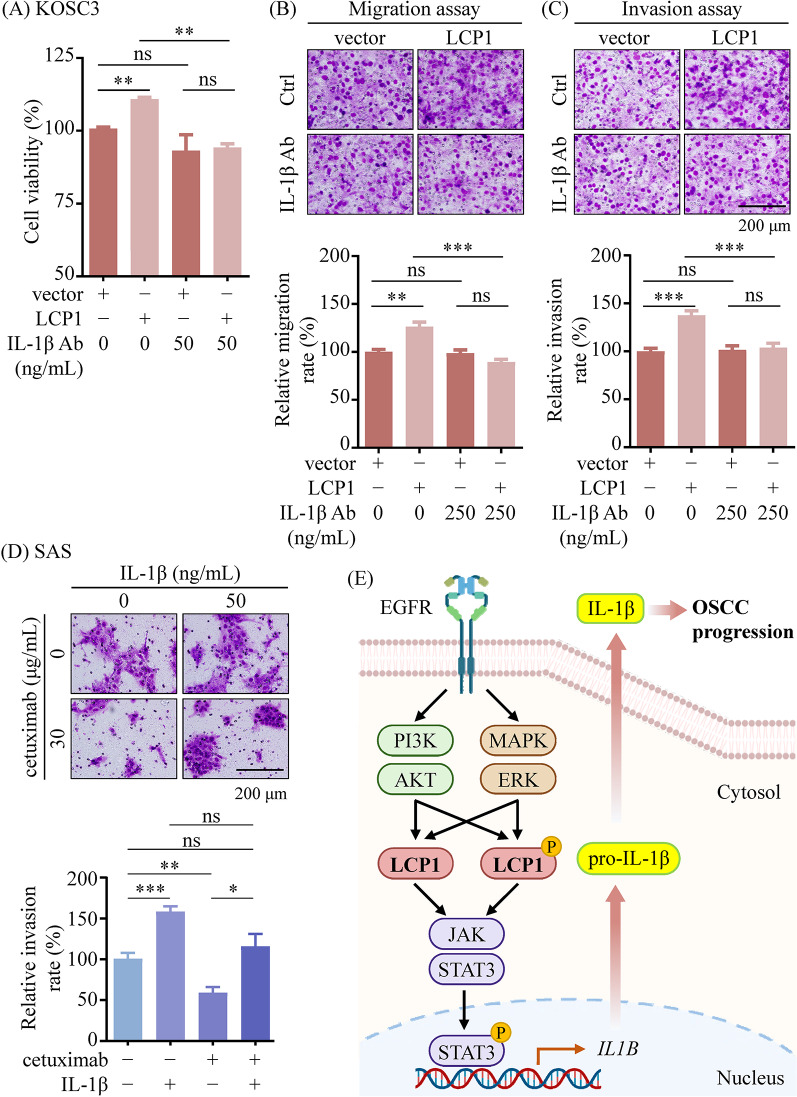
LCP1 enhanced OSCC cell growth and migration through promoting IL-1β secretion. OECM-1 cells stably overexpressing LCP1 were cultured in serum-free media. After 48 h, the conditioned media were collected and used as cultured media of KOSC3 cells, in which LCP1 had been stably knocked down. The proliferation (**A**), migration (**B**) original magnification, ×400; scale bar: 200 μm), and invasion (**C**) original magnification, ×400; scale bar: 200 μm) potentials were examined in the LCP1-knockdown KOSC3 cells with and without IL-1β antibody (Ab) treatment. (**D**) After 24-h serum starvation, SAS cells were treated with EGFR inhibitor cetuximab (30 µg/mL) and/or recombinant IL-1β (50 ng/mL). The invasion potentials of the cells were examined (original magnification, ×400; scale bar: 200 μm). Cell viability and mobility were quantified and presented in the bar graph. Statistical significance was determined by one-way ANOVA. *p < 0.05, **p < 0.01, ***p < 0.001. (**E**) The schematic illustrates the proposed mechanism by which LCP1 promotes proliferation and migration in OSCC cells. The EGFR signaling pathway is pivotal in OSCC progression, activating the PI3K/AKT and MAPK/ERK cascades. These activations upregulate LCP1 expression and its phosphorylation at Ser5, contributing to cisplatin resistance. Furthermore, LCP1 expression activates a JAK2/STAT3 axis to enhance pro-IL-1β expression and subsequent IL-1β secretion, thereby promoting cell growth, mobility, and invasion in OSCC cells

To further validate IL-1β’s role, we treated LCP1-knockdown KOSC3 cells with conditioned media from LCP1-overexpressing OECM-1 cells. This treatment enhanced proliferation, migration, and invasion abilities of the KOSC3 cells, while neutralization with an IL-1β antibody attenuated these effects (Figs. 9A-C). To confirm the involvement of IL-1β in EGFR signaling, we performed a rescue experiment by supplementing EGFR inhibitor (cetuximab)-treated SAS cells with recombinant IL-1β. As shown in Fig. 9D, exogenous IL-1β restored the invasion capacities that were suppressed by EGFR inhibition, indicating that IL-1β is a critical mediator of LCP1-driven OSCC aggressiveness.

In conclusion, our findings reveal that LCP1, upregulated by EGFR signaling, promotes OSCC cell proliferation, migration, and invasion through the JAK2/STAT3/IL-1β axis. Rescue experiments with recombinant IL-1β further confirm its role as a downstream effector in this pathway (Fig. 9E).

## Discussion

The 5-year survival rate for patients with OSCC is approximately 50%, largely due to tumor metastasis and recurrence following treatment [3, 4]. Identifying molecular drivers of OSCC progression is therefore crucial for developing more effective treatment strategies. In this study, we performed comparative proteomic profiling of primary and relapsed OSCC tissues using iTRAQ-based mass spectrometry, revealing distinct protein expression patterns between treatment-naïve and recurrent tumors. Notably, only 8 (12.7%) upregulated proteins overlapped between primary and recurrent OSCC tissues, suggesting that recurrence involves unique biological mechanisms (Figs. 1 C, S1C, Table S6, and S7).

The 8 proteins upregulated in both primary and recurrent OSCC tissues (Fig. 1C) are acidic leucine-rich nuclear phosphoprotein 32 family member B (ANP32B), laminin subunit β1 (LAMB1), nicotinamide N-methyltransferase (NNMT), tenascin (TNC), protein SET (SET), protein enabled homolog (ENAH), cartilage-associated protein (CRTAP), and prolyl 3-hydroxylase 1 (P3H1). Previous studies have documented elevated expression levels of ANP32B, LAMB1, NNMT, TNC, and ENAH in OSCC tissues [44–48]. Notably, NNMT and ENAH have been shown to enhance the proliferative and tumorigenic potential of oral cancer cells [44, 49], while TNC contributes to an immune-suppressive tumor microenvironment in OSCC [50]. SET has been implicated in promoting tumor growth and conferring resistance to cisplatin-induced apoptosis in a HNSCC xenograft model [51]. In contrast, the roles of CRTAP and P3H1 in OSCC progression remain poorly characterized. These findings underscore the utility of parallel proteomic profiling of primary and recurrent tumor tissues as a powerful approach for uncovering molecular drivers of OSCC progression.

Among the proteins with elevated levels in recurrent OSCC tissues, LCP1 stood out due to its association with poor survival and its known role in cytoskeletal remodeling. Our data demonstrate that LCP1 expression and phosphorylation are regulated by EGFR signaling via PI3K/AKT and ERK pathways, enhancing OSCC cell proliferation, migration, and invasion. These findings align with previous reports showing that LCP1 phosphorylation at Ser5, induced by the PI3K/SGK and ERK/MAPK/RSK cascades, promotes actin bundling [35, 52] and LCP1 recruitment to migratory structures in breast cancer cells [22, 53]. Whether EGFR-mediated LCP1 phosphorylation involves SGK or RSK in OSCC remains to be elucidated.

Importantly, our study reveals a novel mechanism by which LCP1 promotes IL-1β production via activation of the JAK2/STAT3 axis. IL-1β is a key inflammatory cytokine implicated in OSCC progression [41, 54], epithelial-to-mesenchymal transition (EMT) induction [55], lymphatic metastasis [56], and TME immune modulation [57]. Prior studies have shown that LCP1 enhances NLRP3 inflammasome activity in macrophages, contributing to IL-1β maturation [39]. Moreover, LCP1 has been linked to immune cell infiltration and inflammatory signaling in various cancers, including triple-negative breast cancer [21], and ischemic brain injury, where it modulates macrophage function and cytokine production [58]. Our findings extend this role to OSCC cells, demonstrating that LCP1 upregulates pro-IL-1β independently of NF-κB activation and that IL-1β promotes OSCC cell aggressiveness in both direct stimulation and conditioned media experiments.

Despite these insights, several limitations should be acknowledged. First, our study did not functionally assess how LCP1 and IL-1β affect immune cell populations within the OSCC TME, which play critical roles in disease progression. Tumor-associated macrophages (TAMs), myeloid-derived suppressor cells (MDSCs), and regulatory T cells (Tregs) contribute to immunosuppression and metastasis in OSCC through cytokine signaling and metabolic reprogramming [59–61]. Future studies should employ single-cell transcriptomics or spatial proteomics to elucidate how LCP1 modulates immune cell dynamics within the TME. Second, although our in vitro data support the involvement of LCP1–IL-1β axis, in vivo validation using OSCC relapse models is essential to confirm its role in tumor recurrence and immune modulation.

Clinically, our findings highlight EGFR and STAT3 as druggable nodes within the LCP1 signaling cascade. EGFR is overexpressed in over 90% of HNSCC cases and is associated with poor prognosis and recurrence [62]. STAT3, a key transcription factor downstream of JAK2, is constitutively activated in OSCC and drives proliferation, invasion, and immune evasion [63–65]. Several inhibitors targeting EGFR (e.g., cetuximab) and STAT3 (e.g., WP1066 and HJC0152) have shown promise in preclinical OSCC models [64, 66]. Our data suggest that targeting the EGFR–LCP1–STAT3–IL-1β axis may offer a novel strategy for treating recurrent OSCC.

In conclusion, this study identifies LCP1 as a critical mediator of OSCC progression through its regulation of EGFR signaling and IL-1β production. By linking cytoskeletal remodeling to inflammatory signaling, LCP1 emerges as both a potential biomarker and a therapeutic target in OSCC. Future studies should aim to validate these findings in clinical cohorts and evaluate combination therapies that disrupt this oncogenic axis.

## Supplementary Information

Supplementary Materials and Methods

Supplemental Tables S1-S3 and S9

Supplemental Table S4-1

Supplemental Table S4-2

Supplemental Table S4-3

Supplemental Table S4-4

Supplemental Table S4-5

Supplemental Table S4-6

Supplemental Table S4-7

Supplemental Table S5

Supplemental Table S6

Supplemental Table S7

Supplemental Table S8

Supplemental Figures

## Data Availability

All data generated or analyzed during this study are included in this published article and its supplementary information files. Original immunoblots are also provided in the supplementary information file. The MS raw data for proteome analysis were deposited on the ProteomeXchange Consortium website (http://proteomecentral.proteomexchange.org) via the PRIDE partner repository, data set identifier: PXD046780.
