# Supplementary material for: LCP1 upregulation via EGFR signaling promotes oral cancer progression through the JAK2/STAT3/IL-1β axis
Source: Cancer Cell Int. 2025 Oct 3;25:329. doi: 10.1186/s12935-025-03970-0 (PMC12495854; doi:10.1186/s12935-025-03970-0)
Supplement: Supplementary file 1 — Supplementary Materials and Methods [file 12935_2025_3970_MOESM1_ESM.pdf]

LCP1 upregulation via EGFR signaling promotes oral cancer progression through the JAK2/STAT3/IL-1β axis

Chiao-Rou Liu, Kai-Ping Chang, Xiu-Ya Chan, Chu-Mi Hung, Chia-Yu Yang, Kuan-Ming Lai, Hao-Ping Liu*, Chih-Ching Wu*

* Correspondence: Chih-Ching Wu, Department of Medical Biotechnology and Laboratory Science, College of Medicine, Chang Gung University, Taoyuan 33302, Taiwan. e-mail: luckywu@mail.cgu.edu.tw. Hao-Ping Liu, Department of Veterinary Medicine, College of Veterinary Medicine, Taichung 402202, Taiwan. e-mail: hpliu@dragon.nchu.edu.tw.

Supplemental Materials and Methods

Patient populations and clinical specimens

Tissues for proteome analysis were collected from 6 treatment-naïve OSCC patients (#1–#6) and 4 patients with relapsed OSCC (#5R, #6R, #7R, and #8R). Notably, #5R and #6R correspond to patients #5 and #6 who experienced relapses post-treatment. For LCP1 qRT-PCR analysis, tissues were harvested from 224 pretreatment OSCC patients. Patient characteristics and clinical features for both the proteomics and qRT-PCR analyses are detailed in **Table 1** and **Supplemental Table S7**, respectively. All specimens were obtained from Linkou Chang Gung Memorial Hospital (CGMH), Taoyuan, Taiwan, between 2015 and 2020. This study was approved by the Institutional Review Board (IRB) of Linkou CGMH and conducted in accordance with the Declaration of Helsinki (Protocol No. 201800700B0 and

102-5685A3). All participants provided informed consent prior to sample collection. Biopsy-confirmed OSCC cases were diagnosed through oral mucosal screening, and patients underwent routine check-ups following standard protocols.

Extraction and tryptic digestion of tissue proteins for iTRAQ labeling

Tissue pieces (approximately 3×3×3 mm^3) from cancerous and adjacent noncancerous areas were flash-frozen in liquid nitrogen and homogenized with 300 μL of RapiGest™ SF (0.1%, Waters Corporation, Milford, MA, USA) using a bead-beater homogenizer (Precellys 24; Bertin Technologies, Ozyme, France). The tissue samples were centrifuged at 14,000 rpm for 10 min at 4°C, and the supernatants were collected and stored at -80°C until further use.

For mass spectrometry analysis, 10 μg of proteins from each sample were reduced with 300 mM triethylammonium bicarbonate (TEABC, Sigma-Aldrich, St. Louis, MO, USA) containing 250 mM tris-(2-carboxyethyl)-phosphine hydrochloride (Sigma-Aldrich) at 60°C for 60 min, followed by alkylation with 300 mM TEABC containing 200 mM S-methyl methanethiosulfonate (Sigma-Aldrich) at room temperature for 30 min. The proteins were then digested with modified sequencing-grade porcine trypsin (Promega, Madison, WI, USA) at 37°C for 16 h.

To quantitatively profile tissue proteomes, the resulting tryptic peptides were labeled with iTRAQ reagents (AB Sciex, Foster City, CA, USA) according to the manufacturer's protocol.

Briefly, the iTRAQ reagent was dissolved in ethanol, mixed with the peptide mixture, and shaken at room temperature for 1 h. Peptides from cancerous and adjacent noncancerous tissues of pretreatment patients (#1–#6) were labeled with iTRAQ 115 and 114 tags, respectively. Peptides from relapsed cancerous and noncancerous tissues of patients (#5R–#8R) were labeled with iTRAQ 117 and 116 tags, respectively.

The iTRAQ-labeled samples were frozen, lyophilized to dryness using Centrifugal Vacuum Concentrators (Labconco, Kansas City, MO, USA), and reconstituted in 0.1% trifluoroacetic acid (TFA). Samples from the same patients were mixed in equal amounts, while samples from #7R and #8R were pooled. The mixed samples were then desalted using a Ziptip with C18 resin (2 μL; GE Healthcare Life Sciences, UK). The peptides were subsequently eluted with 1% TFA containing 30%, 50%, and 70% acetonitrile (ACN). The eluted peptides were dehydrated using a centrifugal vacuum concentrator and stored at -20°C.

Peptide fractionation and mass spectrometry (MS) analysis

The iTRAQ-labeled peptides were separated using online 2-dimensional liquid chromatography (LC; UltiMate™ 3000 RSLCnano System, Thermo Fisher Scientific, San Jose, CA, USA) and analyzed as previously described [1]. Briefly, the peptides were reconstituted in 50 μL of buffer A [30% ACN containing 0.1% formic acid (FA)] and loaded onto an SCX-HPLC column (Luna 5 μm SCX 100 Å, 0.5 × 200 mm, Phenomenex, Taipei, Taiwan) at a flow

rate of 1 μL/min. The peptides were then eluted with a 5-99% gradient of buffer B (30% ACN containing 0.1% FA and 1.0 M ammonium chloride). The resulting fractions were diluted in-line prior to being trapped on a Zorbax 300SB-C_{18} column (0.3 × 5 mm; Agilent Technologies, Wilmington, DE, USA). Each fraction was further separated on a homemade column (HydroRP 2.5 μm, 75 μm inner diameter, 20 cm length) with a 15-μm tip using buffer C (ACN containing 0.1% FA), applying a linear gradient of 6-95% over 60 min at a flow rate of 0.25 μL/min.

The LC system was coupled to an LTQ-Orbitrap Elite mass spectrometer (Thermo Fisher Scientific) operated with Xcalibur software (version 2.2 SP1.48, Thermo Fisher Scientific). Full-scan MS was performed in the Orbitrap over a range of 350 to 2000 Da with a resolution of 60,000 at *m/z* 400. Ion signals of $(Si(CH_3)_2O)_6H^+$ at *m/z* 445.120025, 462.146574, and 536.165365 were used for lock masses and internal calibration. Data-dependent MS/MS scan events were performed, including 6 collision-induced dissociations (CIDs) and 6 higher-energy CIDs (HCDs), followed by one MS scan for the 6 most abundant ions in the preview MS scan. The selected *m/z* values were excluded dynamically for 180 s with a relative mass window of 1.5 Da. The electrospray voltage was set to 1.8 kV, and the capillary temperature was maintained at 220°C. Automatic gain control was applied to prevent ion trap overfilling, with maximum accumulation time/ions set at 1000 ms/2 × 10^6 ions for the full scan, 150 ms/5 × 10^3 ions for CID, and 300 ms/3 × 10^4 ions for HCD.

Protein database searching and quantification

Protein database searching and iTRAQ-based quantification were conducted using Proteome Discoverer software (version 1.4.1.14; Thermo Fisher Scientific). The MS/MS spectra were searched against the Swiss-Prot human sequence database (released in March 2023, specific to *Homo sapiens*, containing 20,426 entries) using the Mascot search engine (version 2.2.0; Matrix Science, London, UK). Mass tolerances were set at 10 ppm for intact peptide masses, 0.5 Da for CID fragment ions, and 0.05 Da for HCD fragment ions, allowing for one missed tryptic cleavage. Fixed modifications included methylthio (Cys, +46.0916 Da) and iTRAQ tags (N-terminus or Lys, +144.1544 Da), while oxidation of Met (+15.9994 Da) was treated as a variable modification.

To estimate the false discovery rate (FDR), spectra were also searched against a decoy database. The FDR was calculated by dividing the number of hits in the decoy database by those in the target database. To maintain an overall FDR < 0.01, peptide confidence criteria were set as a p value < 0.01, peptide length > 7 amino acids, and ≥ 2 peptide hits per protein. Epithelial keratin identifications were excluded from the analysis.

For iTRAQ-based protein quantification, quantitative data were exported from Proteome Discoverer into Excel. Proteins with more than 2 quantifiable spectra were considered quantifiable. The iTRAQ ratios were transformed to a $\log_2$ scale and manually normalized so that the $\log_2$ protein ratios displayed a median value of zero. Proteins with $\log_2$ ratios exceeding

the mean plus one standard deviation (SD) were considered overexpressed, while those with log_2 ratios below the mean minus one SD were considered underexpressed.

Bioinformatics analysis

Functional enrichment analysis of dysregulated proteins in OSCC tissues was conducted using GO term and biological pathway analyses via STRING (version 11.5). Data visualization and graphing of biological processes and Reactome pathways were performed using SRplot (http://www.bioinformatics.com.cn/SRplot) [2].

For transcriptome analysis of oral cancer tissues, RNA sequencing (RNA-Seq) data from 310 OSCC patients were obtained from the TCGA database through cBioPortal (https://www.cbioportal.org/). Gene expression was calculated using RSEM and batch normalized from Illumina HiSeq_RNASeqV2 data. Survival analysis was conducted based on Z-scores of mRNA expression in OSCC patients. Gene expression correlations between LCP1 and other genes of interest were determined using RNA-Seq data from Taiwanese OSCC patients [3].

Cell culture and transfection

OSCC cell lines KOSC3 and SAS were cultured in medium supplemented with 10% fetal bovine serum (FBS; Gibco, Waltham, MA, USA) and 1% penicillin/streptomycin solution (P/S;

Gibco). KOSC3 cells were maintained in Roswell Park Memorial Institute 1640 medium (RPMI-1640; Gibco), while SAS cells were grown in Dulbecco's modified Eagle's medium (DMEM; Gibco). Both cell lines were incubated at 37°C with 5% CO_2.

For mechanistic studies, epidermal growth factor (EGF; Cat. No. 324831; Merck Taipei, Taiwan) was used to activate EGF receptor (EGFR) signaling, while GSK1059615 (Cat. No. 11569; Cayman Chemical, Ann Arbor, Michigan, USA) and U0126 (Cat. No. 70970; Cayman Chemical) were utilized to inhibit PI3K and ERK1/2, respectively. Cisplatin (Cat. No. p4394; Sigma-Aldrich) was used to suppress OSCC cell growth.

LCP1 expression in KOSC3 cells was inhibited using LCP1 smart pool siRNA (Cat. No. L-011716-00-0005, Dharmacon, Lafayette, CO, USA) transfected with Lipofectamine RNAiMAX (Cat. No. 13778150; Invitrogen, Grand Island, NY, USA). ON-TARGETplus Non-targeting Control siRNA (Cat. No. D001810-10-20, Dharmacon) served as the control. For LCP1 overexpression, the LCP1-coding sequence was inserted into the pcDNA3.1C plasmid, and transfection into SAS cells was performed using TransIT-X2 Transfection Reagent (Cat. No. MR-MIR6000, Mirus Bio, Madison, WI, USA).

MTT cell proliferation assay

OSCC cells (5×10^3 cells/well for KOSC3 and 10^4 cells/well for SAS) were seeded in 96-well plates and maintained at 37°C with CO_2. At the specified time points, the culture media

were removed, and the cells were treated with media containing 10% 3-(4,5-dimethylthiazol-2-yl)-2,5-diphenyl tetrazolium bromide (MTT; Cat. No. M6494, Invitrogen) for 1 h at 37°C. After the supernatant was discarded, the resulting formazan crystals were dissolved in dimethyl sulfoxide (DMSO), and absorbance was measured at 540 nm using a spectrophotometer.

Cell migration, invasion, and wound healing assays

For wound healing assays, OSCC cells (1.8×10^4 and 3.2×10^4 cells/well for KOSC3 and SAS, respectively) were seeded into Culture-Insert 4 Well (Cat. No. 80469, ibidi LLC, Verona, WI, USA) 24 h post-transfection. The culture-insert was removed at the indicated time points, and the gap areas were quantified using Image J software.

For transwell migration and invasion assays, KOSC3 and SAS cells (1.5×10^5 cells/chamber) were seeded into Transwell Polyester Membrane Inserts (Cat. No. 3464, Corning, Taipei, Taiwan). Migration assays were performed without Matrigel (Cat. No. 354234, Corning) coating, while invasion assays used Matrigel-coated inserts. Cells were suspended in 200 μL of culture medium containing 0.5% FBS in the upper chamber, with 700 μL of culture medium containing 30% FBS in the lower chamber. After 24-h incubation, the migrated or invaded cells were fixed with methanol for 20 min, stained with 20% Giemsa solution for 1 h, and counted in 10 fields of view under a microscope at 200× and 400× magnifications for KOSC3 and SAS cells, respectively.

RNA extraction and quantitative real-time PCR (qRT-PCR)

Total RNA was extracted using the TOOLSmart RNA Extractor (Cat. No. DPT-BD24, Biotools, New Taipei City, Taiwan). cDNA was synthesized with the TOOLS@ Quant II Fast RT Kit (Cat. No. KRT-BA06-2, Biotools). qRT-PCR was performed using the TOOLS@ SYBR Green qPCR Mix (Cat. No. FPT-BB05, Biotools). Primer sequences for qRT-PCR are provided in **Supplemental Table S1**.

Extraction of cellular proteins for Western blot

To assess protein levels via immunoblotting, proteins were extracted from OSCC tissues and cells, separated by 10% SDS-PAGE, and transferred onto PVDF membranes. The membranes were incubated overnight at 4°C with primary antibodies, followed by incubation with HRP-conjugated secondary antibodies. Visualization was performed using ECL substrate (Cat. No. WBKLS0500, Merck), and the luminescence signals were detected with a Biostep Chemiluminescence Imager Celvin S 420 (Biostep, Burkhardtsdorf, Germany). The primary antibodies used included rabbit anti-LCP1 (1:3000; Cat. No. 13025-1-AP, Proteintech, Chicago, IL, USA), phospho-LCP1 (Ser5) (1:1000; Cat. No. 12455-1, Signalway Antibody, Greenbelt, Maryland, USA), rabbit anti-AKT (1:1000; Cat. No. 9272), phospho-AKT (Ser473) (1:1000; Cat. No. 9271), rabbit anti- ERK1/2 (1:1000; Cat. No. 4695), phospho-ERK1/2

(Thr202/Tyr204) (1:1000; Cat. No.9101), mouse anti-STAT3 (1:1000; Cat. No. 9139), phospho-STAT3 (Tyr705) (1:1000; Cat. No. 9145), mouse anti-IL-1β (1:500; Cat. No. 12242; all from Cell Signaling, Danvers, MA, USA), mouse anti-GAPDH (1:5000; Cat. No. sc-32233, Santa Cruz Biotechnology, Santa Cruz, CA, USA), and mouse anti-β-actin (1:5000; Cat. No. MAB8929, R&D Systems, Minneapolis, MN, USA).

Statistical analysis

Kaplan-Meier plots were stratified by the median LCP1 expression, with *p*-values obtained using the Gehan-Breslow-Wilcoxon test. Differences in *LCP1* gene expression between noncancerous and tumor tissues in OSCC patients were assessed with paired *t*-tests, while differences between disease-free and recurrent groups were evaluated using unpaired *t*-tests. For qRT-PCR, proliferation, migration, invasion, and wound healing assays, between-group comparisons were performed using Student's *t*-tests or one-way ANOVA. Correlations between *LCP1* and genes of interest were determined using Pearson's correlation coefficient. Each experiment was repeated three times, and consistent trends across the replicates were required for conclusions. Data presented are from one of the three replicates. Statistical tests were two-sided, with a p-value < 0.05 considered statistically significant. All analyses were conducted using Prism software (version 9.0, GraphPad Software, La Jolla, CA, USA).

References

1. Chu, H.W., Chang, K.P., Hsu, C.W., Chang, I.Y., Liu, H.P., Chen, Y.T., and Wu, C.C. (2019). Identification of salivary biomarkers for oral cancer detection with untargeted and targeted quantitative proteomics approaches. Mol Cell Proteomics *18*, 1796-1806. 10.1074/mcp.RA119.001530.
2. Tang, D., Chen, M., Huang, X., Zhang, G., Zeng, L., Zhang, G., Wu, S., and Wang, Y. (2023). SRplot: A free online platform for data visualization and graphing. PLoS One *18*, e0294236. 10.1371/journal.pone.0294236.
3. Yang, C.Y., Liu, C.R., Chang, I.Y., OuYang, C.N., Hsieh, C.H., Huang, Y.L., Wang, C.I., Jan, F.W., Wang, W.L., Tsai, T.L., et al. (2020). Cotargeting CHK1 and PI3K synergistically suppresses tumor growth of oral cavity squamous cell carcinoma in patient-derived xenografts. Cancers (Basel) *12*, 1726. 10.3390/cancers12071726.
