# Supplementary material for: LCP1 upregulation via EGFR signaling promotes oral cancer progression through the JAK2/STAT3/IL-1β axis
Source: Cancer Cell Int. 2025 Oct 3;25:329. doi: 10.1186/s12935-025-03970-0 (PMC12495854; doi:10.1186/s12935-025-03970-0)
Supplement: Supplementary file 2 — Supplemental Tables S1-S3 and S9 [file 12935_2025_3970_MOESM2_ESM.pdf]

Supplemental Table S1. Clinicopathological characteristics of OSCC tissues used for proteome analysis

Patient ID	Sex	Age (years)	TNM classification	Overall stage	Cell differentiation	Treatment after surgery[a]	
						primary tumor	relapsed tumor
Primary tumor							
#1	male	55	T2 N0 M0	2	well	-	-
#2	male	67	T4A N0 M0	4A	moderate	-	-
#3	male	40	T2 N0 M0	2	moderate	RT	-
#4	male	49	T3 N1 M0	4	moderate	CCRT	-
#5	male	52	T3 N0 M0	3	moderate	CCRT	-
#6	male	64	T2 N2B M0	4A	well	CCRT	-
Relapsed tumor							
#5R[b]	male	55	T2 N0 M0	2	moderate	CCRT	CCRT
#6R[b]	male	71	T4A N0 M0	4A	moderate	CCRT	CCRT
#7R	male	57	T2 N0 M0	2	moderate	RT	-
#8R	male	56	T4A N0 M0	4A	well	RT	CCRT

[a] -, no treatment after surgery; RT, radiation therapy; CCRT, concurrent chemo-radiotherapy.

[b] The patients #5R and #6R represent the patients #5 and #6 who developed relapses after treatments.

Supplemental Table S2. List of primers used in qRT-PCR analysis

Gene	Forward primer	Reverse primer
LCP1	AACCAGAGAACCAGGACATTG	AGGGCATCTGATAAGTCACTGT
IL1β	AAACAGATGAAGTGCTCCTTCC	GTCCATGGCCACAACAACT
IL18	TGTAGAGATAATGCACCCCGGA	TGTTCTCACAGGAGAGAGTTGA
TBP	TGCTCACCCCACCAACAATTTAG	CTGGGTTTGATCATTCTGTAGATTAA
ACTIN	TCCACCTTCCAGCAGATG	GTGTAACGCAACTAAGTCATAG
RPN18S	GCTTAATTTGACTCAACACGGGA	AGCTATCAATCTGTCAATCCTGTC
GAPDH	TCATTTCCTGGTATGACAACGA	TCTCTCTTCCTCTTGTGCTC

Supplemental Table S3. Protein quantified in OSCC tissues with iTRAQ-based MS analysis

Patient ID	No. of protein quantified[a]	T/N ratio of proteins[b]		No. of proteins with increased/ reduced levels in tumor tissues[c]
		Mean	SD	
Primary tumor				
#1	4153	-0.147	1.021	442 / 514
#2	4558	-0.075	0.823	448 / 479
#3	4991	-0.066	0.876	464 / 534
#4	5199	-0.095	0.776	539 / 531
#5	4494	-0.083	0.700	505 / 522
#6	4570	-0.141	1.059	518 / 681
Relapsed tumor				
#5R	4541	-0.087	0.707	347 / 445
#6R	4570	-0.061	0.740	492 / 557
#7R	4776	-0.098	0.723	497 / 585
#8R	4808	-0.078	0.727	459 / 560

[a] Number of proteins identified with at least 2 unique peptides and quantified with more than 2 iTRAQ spectra.

[b] Protein ratio in tumor (T) to noncancerous (N) tissues were transformed into $\log_2$ scale. The mean and standard deviation (SD) for $\log_2$ T/N ratios of all proteins in each comparison were acquired.

[c] Proteins with T/N ratios larger than the mean + SD were considered up-regulated, whereas proteins with T/N ratios less than the mean - SD were deemed down-regulated.

Supplemental Table S9. Associations between clinicopathological characteristics and LCP1 expression in tumor tissues from 224 patients with OSCC

Characteristics	No. of patients	No. (percentage) of patients		p-value[b]
		Low expression of LCP1[a]	High expression of LCP1[a]	
Age (years)				
≤ 52.7	112	56 (50.0%)	56 (50.0%)	1.000
> 52.7	112	56 (50.0%)	56 (50.0%)	
Sex				
Male	208	102 (49.0%)	106 (51.0%)	0.299
Female	16	10 (62.5%)	6 (43.6%)	
Tumor classification				
T1-T2	91	37 (40.7%)	54 (59.3%)	0.021
T3-T4	133	75 (56.4%)	58 (43.6%)	
Node classification				
N=0	114	57 (50.0)	57 (50.0%)	1.000
N>0	110	55 (50.0%)	55 (50.0%)	
Overall TNM stage				
I-II	61	27 (44.3%)	34 (55.7%)	0.293
III-IV	163	85 (52.1%)	78 (47.9%)	
Cell differentiation[c]				
Well-moderate	193	100 (51.8%)	93 (48.2%)	0.228
Poor	30	12 (40.0%)	18 (60.0%)	
Tumor depth				
< 10 mm	82	35 (42.7%)	47 (57.3%)	0.086
≥ 10 mm	141	77 (54.6%)	64 (45.4%)	

[a] *LCP1* gene expression in tumor (T) and adjacent noncancerous (N) tissues from 224 patients with OSCC was determined using qRT-PCR. The median T/N ratio of *LCP1* gene expression was used to define low and high expression of *LCP1* gene in OSCC tissues.

[b] The p value was determined by chi-square analysis.

[c] Well-moderate, well-to-moderate differentiation; Poor, poor differentiation.
