# Supplementary material for: LCP1 upregulation via EGFR signaling promotes oral cancer progression through the JAK2/STAT3/IL-1β axis
Source: Cancer Cell Int. 2025 Oct 3;25:329. doi: 10.1186/s12935-025-03970-0 (PMC12495854; doi:10.1186/s12935-025-03970-0)
Supplement: Supplementary file 14 — Supplemental Figures [file 12935_2025_3970_MOESM14_ESM.pdf]

Supplemental Fig. S1

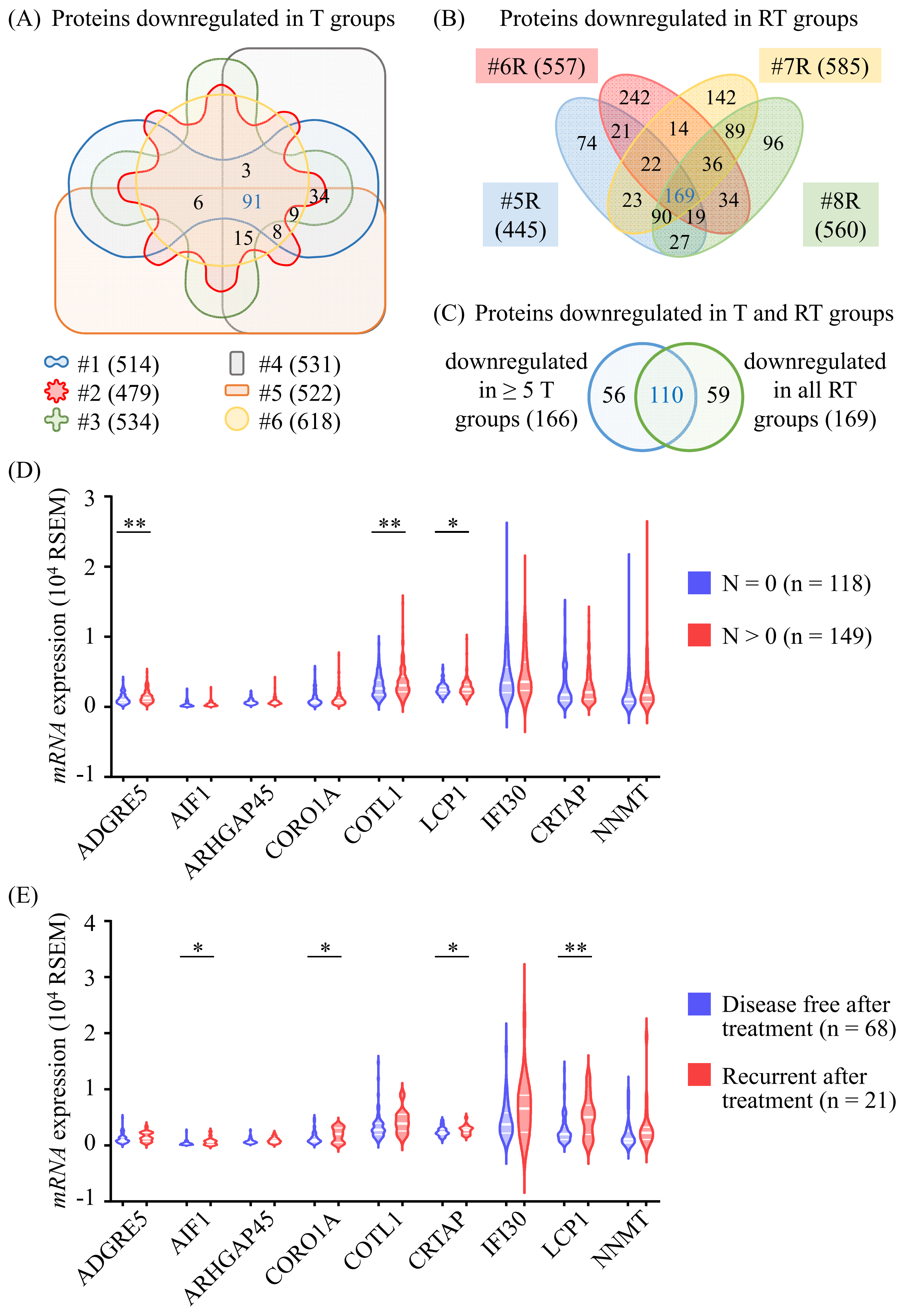

Supplemental Fig. S1

Supplemental Fig. S1. Proteome profiling of tumor tissues collected from patients with primary and relapsed OSCC. Tumor tissues and their case-matched adjacent noncancerous tissues form 6 patients with primary OSCCs (#1, #2, #3, #4, #5, and #6) and 4 patients with relapsed OSCCs (#5R, #6R, #7R, and #8R) were collected. Tissue proteome of primary tumor (T), adjacent noncancerous tissues of T (N), relapsed tumor (RT), and adjacent noncancerous tissues of RT (RN) was analyzed with an iTRAQ-based mass spectrometry. The protein ratios of T/N and RT/RN were acquired. The means and standard deviations (SDs) for the ratios of all proteins in each comparison were obtained. Proteins with ratios below the mean - SD were considered to be downregulated. (A) Venn diagram illustrates the degree of overlap between the proteins downregulated in the T groups. Blue and black numbers indicate the number of proteins with decreased levels in all T groups and 5 of 6 T groups, respectively. (B) Venn diagram shows overlaps between the proteins downregulated in the RT groups. (C) Venn diagram displays overlap between the proteins downregulated in the T and RT groups. The total numbers of identified proteins are listed in brackets. (D, E) The expression profile of genes in OSCC tissues was surveyed using RNA-Seq data from the cBioPortal website. (D) Gene expression was compared in primary tumors with (n = 149) and without (n = 118) lymph node metastasis. (E) Gene expression was compared in primary tumors of OSCC patients who relapsed after treatment (n = 21) and those who were disease-free after treatment (n = 68). Gene expression is displayed with a violin plot, and the white line shows the quartiles of gene expression. Differences between groups were determined using parametric unpaired *t*-tests. *, $p < 0.05$; **, $p < 0.01$.

Supplemental Fig. S2

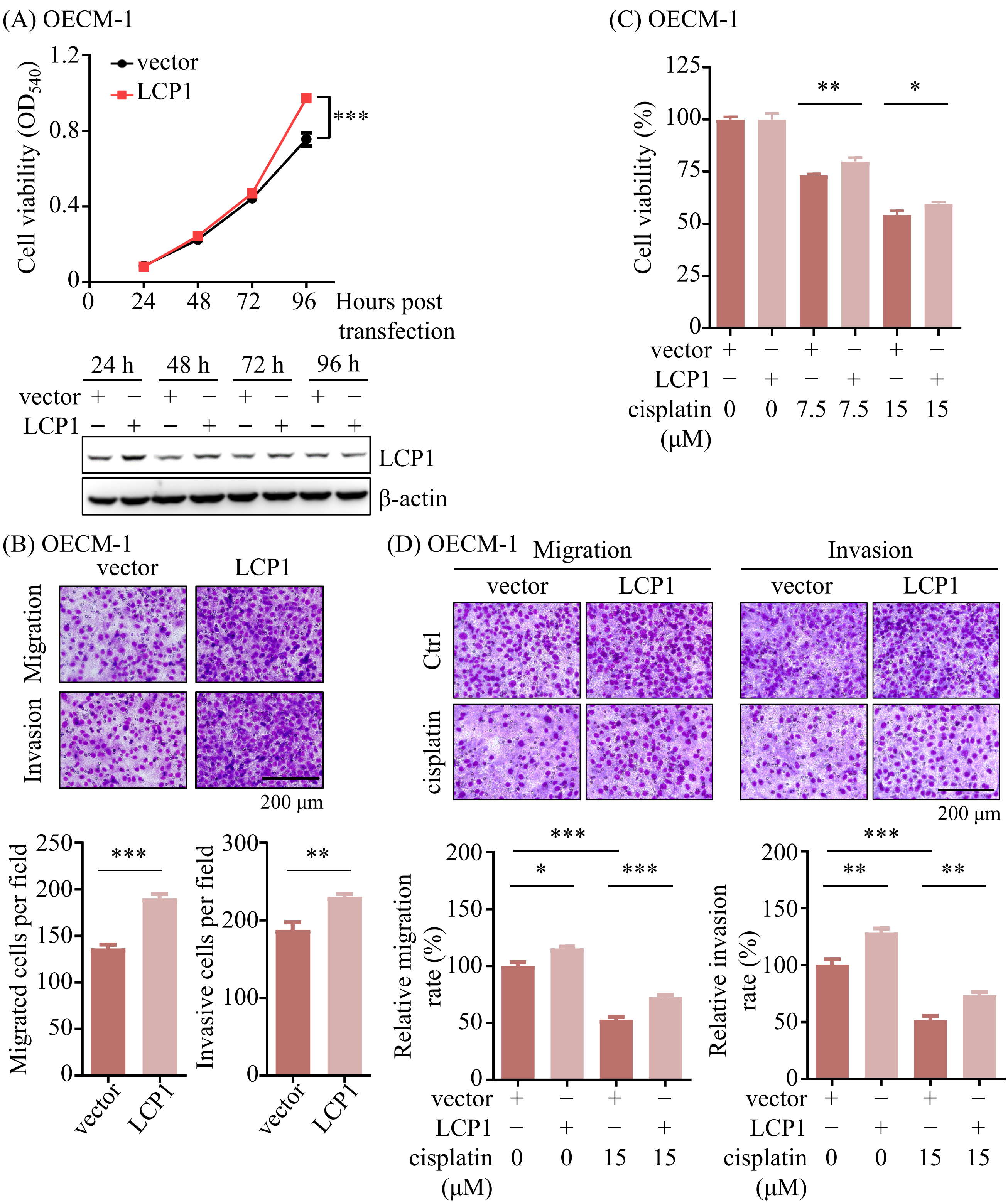

Supplemental Fig. S2. LCP1 overexpression enhances proliferation, migration, and cisplatin resistance in OECM-1 cells. (A) MTT assay for cell proliferation were performed using OECM-1 cells transfected with either a control vector or an LCP1 expression plasmid. (B) Transwell migration and Matrigel invasion assays (original magnification, ×400; scale bar: 200 μm) were conducted to assess the effects of LCP1 overexpression. (C) The proliferation and (D) migration and invasion capabilities of LCP1-overexpressing OECM-1 cells were evaluated with and without cisplatin treatment. Quantification of migration and invasion abilities (B, D) is presented in bar graphs. Statistical significance was determined using parametric unpaired t-tests (A-C) and one-way ANOVA (D). $*p < 0.05$, $**p < 0.01$, $***p < 0.001$.

Supplemental Fig. S3

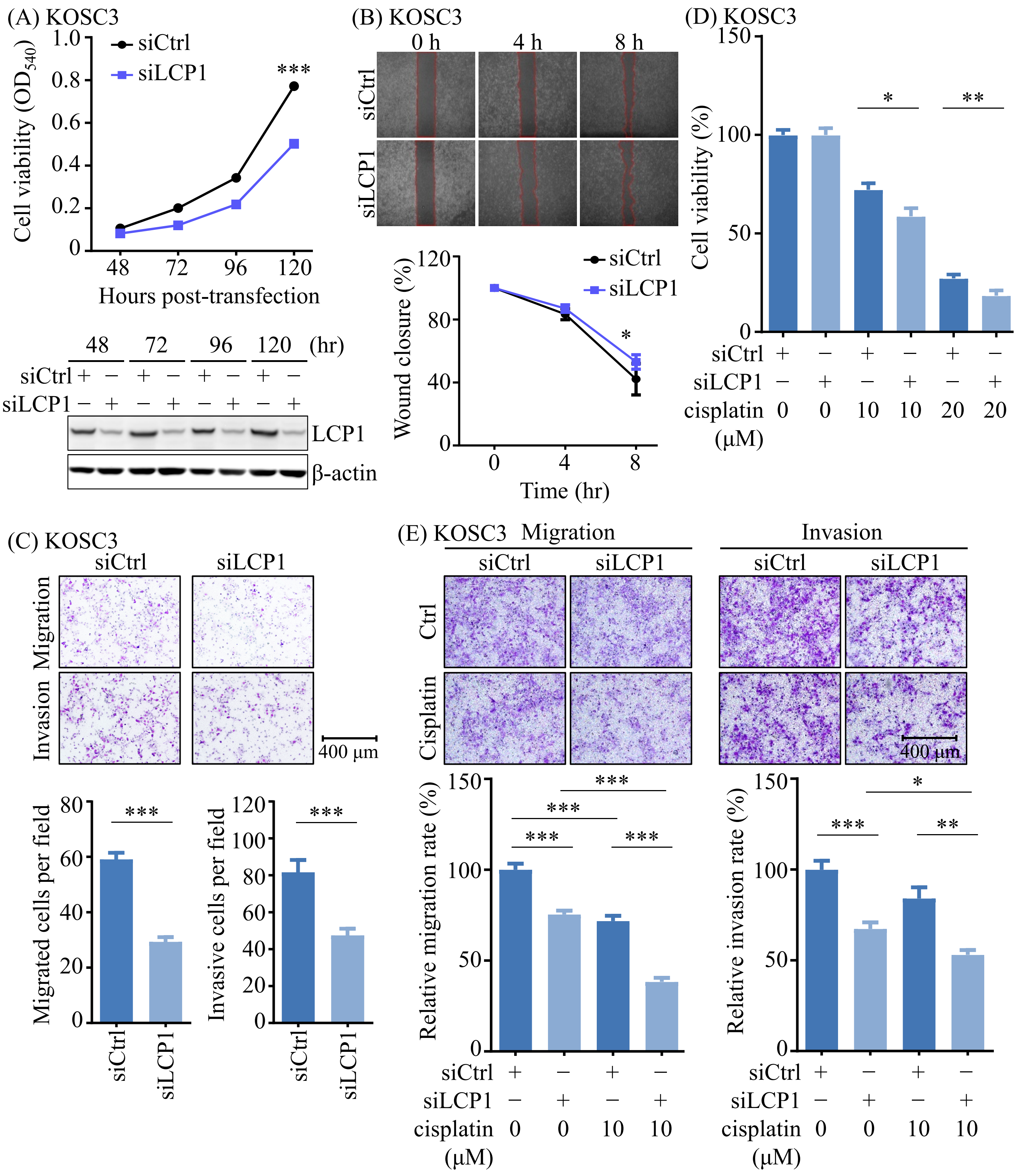

Supplemental Fig. S3. Inhibition of LCP1 expression results in decreased proliferation, migration, and cisplatin tolerance in KOSC3 cells. MTT cell proliferation (A), wound healing (B), transwell migration and matrigel invasion (C; original magnification, ×200; scale bar: 400 μm) assays were performed with the KOSC3 cells transfected with the control siRNA (siCtrl) and the LCP1-specific siRNA (siLCP1). The proliferation (D), migration, and invasion (E; original magnification, ×200; scale bar: 400 μm) abilities of the LCP1-knockdown KOSC3 cells were determined with or without cisplatin treatment. Migration and invasion abilities (C, E) were quantified and shown in the bar graph. The *p*-values were determined by parametric unpaired *t*-tests (A-D) and one-way ANOVA (E). *, $p < 0.05$; **, $p < 0.01$; ***, $p < 0.001$.

Supplemental Fig. S4

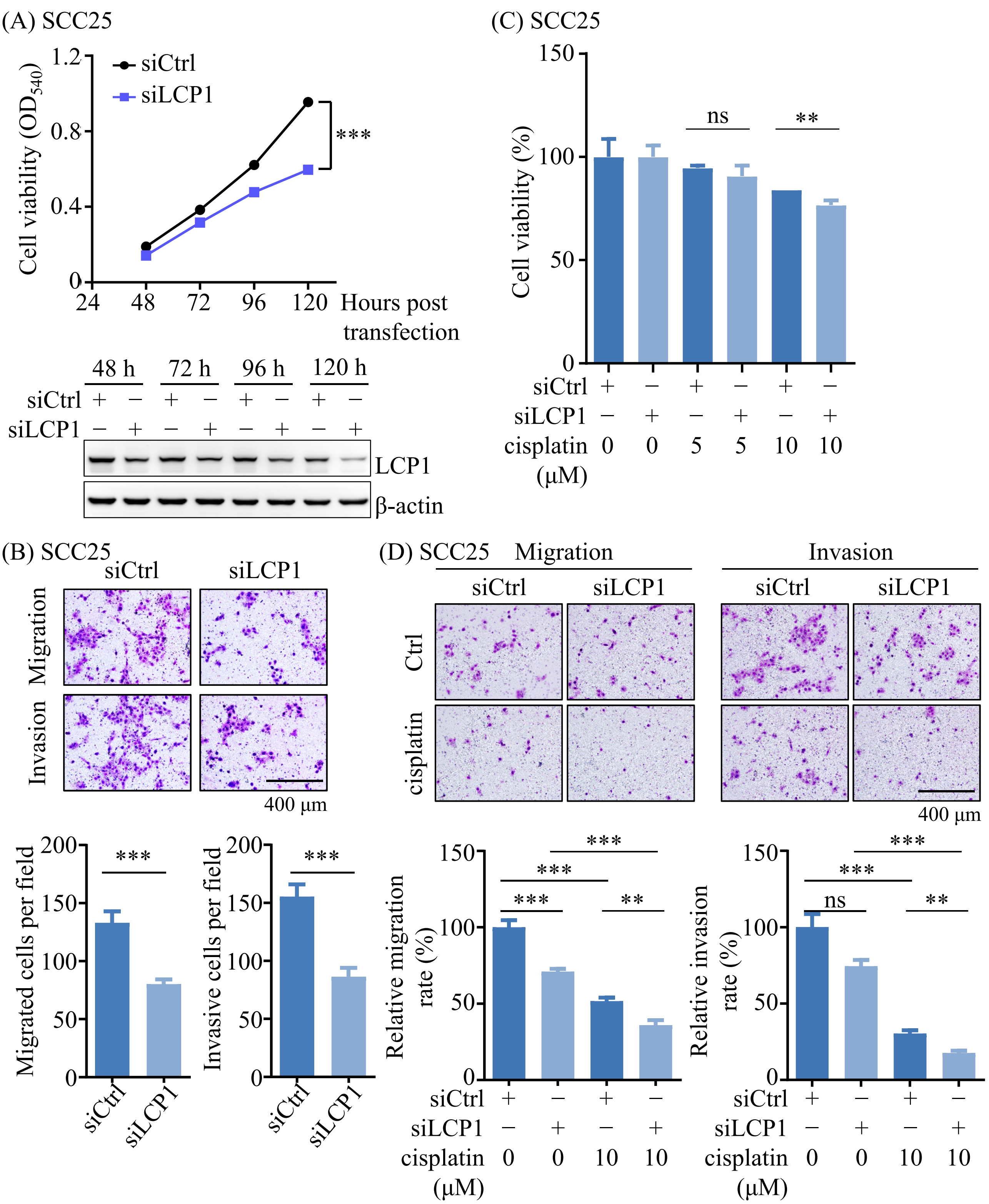

Supplemental Fig. S4. Inhibition of LCP1 expression results in decreased proliferation, migration, and cisplatin tolerance in SCC25 cells. MTT cell proliferation (A), transwell migration and matrigel invasion (B; original magnification, ×200; scale bar: 400 μm) assays were performed with the SCC25 cells transfected with the control siRNA (siCtrl) and the LCP1-specific siRNA (siLCP1). The proliferation (C), migration, and invasion (D; original magnification, ×200; scale bar: 400 μm) abilities of the LCP1-knockdown SCC25 cells were determined with or without cisplatin treatment. Migration and invasion abilities (B, D) were quantified and shown in the bar graph. Statistical significance was determined using parametric unpaired *t*-tests (A-C) and one-way ANOVA (D). *, $p < 0.05$; **, $p < 0.01$; ***, $p < 0.001$.

Supplemental Fig. S5

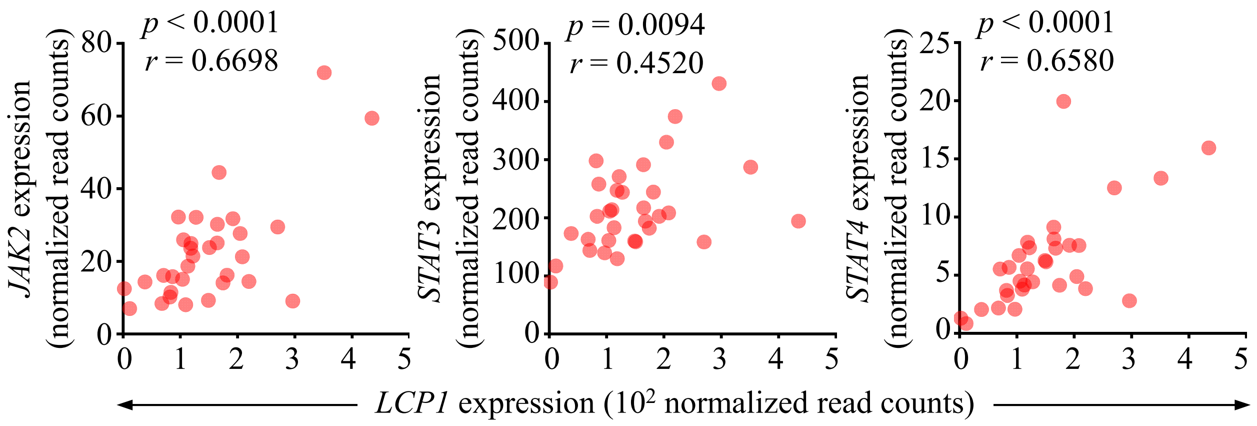

Supplemental Fig. S5. The JAK/STAT axis acts downstream of LCP1 in OSCC cells. The gene expression correlation between LCP1 and STAT in OSCC tissues was examined with the RNA-Seq data (Yen et al., Front Oncol. 2022, 12, 792297), in which the primary OSCC tissues from 32 Taiwanese OSCC patients were included. Pearson correlation was used to measure the association between LCP1 and candidate genes.

Supplemental Fig. S6

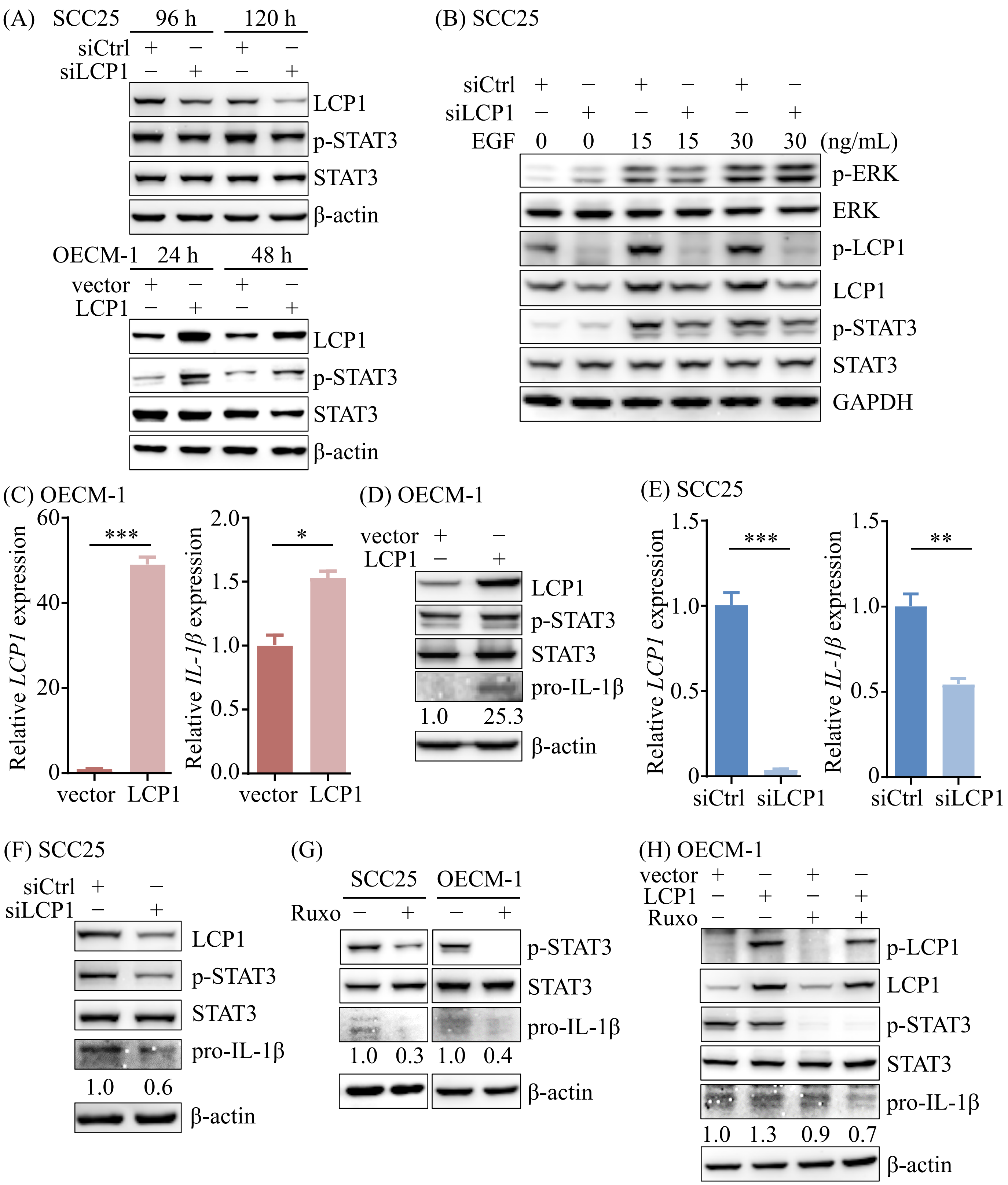

Supplemental Fig. S6

Supplemental Fig. S6. LCP1 upregulates IL-1β via the JAK/STAT3 axis in OSCC cells. (A) Phosphorylation of STAT3 at Tyr705 was assessed in SCC25 cells transfected with control siRNA (siCtrl) or LCP1-specific siRNA (siLCP1), and in OECM-1 cells transfected with either a control vector or an LCP1 expression plasmid. (B) LCP1-knockdown SCC25 cells were treated with EGF for 1 h, and proteins of interest were detected using Western blotting with the indicated antibodies. (C-F) OECM-1 cells were transfected with either a control vector or an LCP1 expression plasmid. After 24 h, LCP1 and pro-IL-1β expression levels were measured using qRT-PCR (C) and Western blotting (D). SCC25 cells were transfected with siCtrl or siLCP1. After 120 h, LCP1 and pro-IL-1β expression levels were measured using qRT-PCR (E) and Western blotting (F). (G) SCC25 and OECM-1 cells were treated with 10 μM JAK1/2 inhibitor ruxolitinib (Ruxo). Proteins of interest were detected with Western blotting after 24 h of ruxolitinib treatment. (H) OECM-1 cells overexpressing LCP1 were treated with 10 μM ruxolitinib for 2 h following 24 h of LCP1 overexpression. Whole-cell lysates were analyzed by immunoblotting with the indicated specific antibodies. Pro-IL-1β levels in experimental groups are presented as fold changes relative to the control group. *ACTN* is used as an internal control gene for the qRT-PCR analyses. Statistical significance was determined by unpaired Student's *t*-tests. *$p < 0.05$. **$p < 0.01$. ***$p < 0.001$.

Supplemental Fig. S7

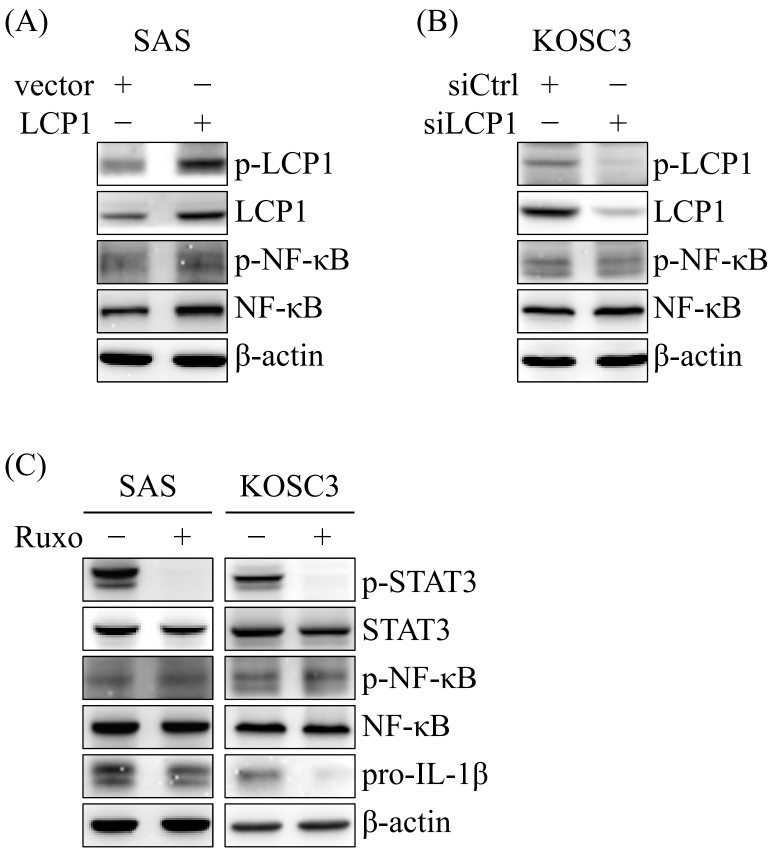

Supplemental Fig. S7. LCP1-mediated activation of NF-κB pathway may be not involved in LCP1-induced IL1β production. (A, B) Protein phosphorylation (LCP1 at Ser5 and NF-κB at Ser536) were detected in the LCP1-overexpressed SAS cells (A) and the LCP1-knockdown KOSC3 cells (B). (C) IL-1β production, NF-κB phosphorylation, and STAT3 phosphorylation at Tyr705 were detected in the OSCC cells treated with the JAK1/2 inhibitor ruxolitinib (Ruxo) for 24 h.
